# Reactive optimal motion planning for a class of holonomic planar agents using reinforcement learning with provable guarantees

**DOI:** 10.3389/frobt.2023.1255696

**Published:** 2024-01-03

**Authors:** Panagiotis Rousseas, Charalampos Bechlioulis, Kostas Kyriakopoulos

**Affiliations:** ^1^ Control Systems Laboratory, School of Mechanical Engineering, National Technical University of Athens, Athens, Greece; ^2^ Division of Systems and Control, Department of Electrical and Computer Engineering, University of Patras, Patras, Greece; ^3^ Center of AI & Robotics (CAIR), New York University, Abu Dhabi, United Arab Emirates

**Keywords:** optimal motion planning, optimal control, reinforcement learning, nonlinear systems and control, path planning

## Abstract

In control theory, reactive methods have been widely celebrated owing to their success in providing robust, provably convergent solutions to control problems. Even though such methods have long been formulated for motion planning, optimality has largely been left untreated through reactive means, with the community focusing on discrete/graph-based solutions. Although the latter exhibit certain advantages (completeness, complicated state-spaces), the recent rise in Reinforcement Learning (RL), provides novel ways to address the limitations of reactive methods. The goal of this paper is to treat the reactive optimal motion planning problem through an RL framework. A policy iteration RL scheme is formulated in a consistent manner with the control-theoretic results, thus utilizing the advantages of each approach in a complementary way; RL is employed to construct the optimal input without necessitating the solution of a hard, non-linear partial differential equation. Conversely, safety, convergence and policy improvement are guaranteed through control theoretic arguments. The proposed method is validated in simulated synthetic workspaces, and compared against reactive methods as well as a PRM and an RRT^⋆^ approach. The proposed method outperforms or closely matches the latter methods, indicating the near global optimality of the former, while providing a solution for planning from anywhere within the workspace to the goal position.

## 1 Introduction

Motion Planning (MP), is one of the most fundamental problems in robotics (Latombe, 1999). Almost any resultant problem (from industry-related tasks, to social and collaborative ones) is irrelevant if a robot fails to autonomously perform safe and correct navigation. While in the industrial context such problems have been largely addressed, owing to highly restricted operating conditions and high task specificity, extending robotic capabilities for tackling any highly unknown, high-variance, and adaptive real-world environment is an active research field in modern robotics.

In order to tackle the above demanding problem, Data Driven methods (DT) and Machine Learning (ML) approaches (and more specifically Reinforcement Learning (RL)) (Brunton and Kutz, 2021) have been employed with remarkable results. Traditionally, robotics problems are treated as Control Theoretic (CT) problems, which are tackled through formal control *design* based on the aforementioned theoretical framework. RL methodologies treat such problems through *learning* a control policy via various methods that in general require exploration of a robot’s environment and the exploitation of gathered knowledge (data). The learning aspect of ML methods aims at addressing an integral feature of robotic platforms, namely, the concept of “Embodied Intelligence” (Roy et al., 2021) (i.e., the fact that embodied agents operate within and interact with realistic, highly varying, and uncertain environments).

However, even though ML researchers have provided some theoretical guarantees (Sutton and Barto, 1998), this rests in contrast to the CT paradigm, where provable guarantees are a design specification (e.g., see feedback linearization (Isidori, 1999) for convergence and prescribed performance control (Bechlioulis and Rovithakis, 2008) for output constraints). For example, in the context of the motion planning problem, there exist a variety of conventional algorithms that provide provable guarantees, whereas ML approachesmostly aim at achieving a desired control performance, without imposing the latter by design, hence, a statistical analysis may be applied to asses the effectiveness of the method. Considering the above, we propose a method that aims at taking a step towards bridging the identified gap in the context of the MP problem. In this case, the two perspectives, namely, the control theoretic reactive (feedback) design and RL-based optimization, are merged via a Policy Iteration (PI) approach. The design of a provably safe and convergent controller follows the CT philosophy, while at the same time, it is shown to provide successively ameliorating policies through the application of a RL-PI scheme. The proposed method concentrates around the off-line computation of the optimal velocity input for a class of holonomic agents, which is advantageous in cases where planning from many initial robot positions is necessary. Such environments include warehouses, where the workspace is *a-priori* known and robotic tasks most likely include many navigation instances within a robot’s workday, thus motivating our method’s scope.

### 1.1 Related work

The Path Planning problem, i.e., the definition of a geometric path given geometric constraints, has been extensively approached using Sampling-Based Methods (SBMs) (Karaman and Frazzoli, 2011) and/or discrete/graph-based approaches. These include, but are not limited to, Probabilistic Road Maps (PRMs) (Kavraki et al., 1996), Djikstra’s algorithm (Anastopoulos et al., 2009), Random Rapidly exploring Trees (RRT) (LaValle et al., 2001) and its variant RRT^⋆^ (Karaman and Frazzoli, 2011), A^⋆^ (Pearl, 1984) and D^⋆^ with its variant, D^⋆^-lite (Koenig and Likhachev, 2002). Owing to the rise in computing power, such methods have been widely celebrated, with significant extensions to improve their performance, or generalize their scope. RRT^⋆^ has more specifically seen significant development, through smart re-planning (Wang et al., 2020b) and extensions such as (Jaillet et al., 2008) for considering continuous cost formulations, and even dynamic workspaces (Wang et al., 2020a).

In contrast to discrete methods, continuous methods, like Navigation Functions (NFs) (Rimon and Koditschek, 1992) and Artificial Potential Fields (APFs), treat the problem through the design of potential functions over the robot’s workspace, such that they exhibit a single global minimum at the desired configuration. However, such methods are hard to tune in order to nullify any local minima that would inhibit convergence to the desired final position. A notable class of APFs, namely, Artifical Harmonic Potential Fields (AHPFs) (Loizou, 2011) have been employed in disk worlds, owing to lack of local minima inside their domain. Their limitations have also been treated through transformations of arbitrary workspaces into disk ones (Vlantis et al., 2018a), and the formulation of harmonic vector basis’ directly on the physical workspace (Kim and Khosla, 1991; Rousseas et al., 2021; Rousseas et al., 2022). The above methods are consistent with the reactive approach of CT, through the formulation of a continuous velocity vector field over the entire workspace of a robot, while providing convergence and safety guarantees for the resulting velocity field.

More recently, ML methods have been employed for a wide range of robotic planning problems, including MP. Long-Short-Term-Memory Networks (LSTMNs) (Inoue et al., 2019), Support Vector Machines (SVMs) (Weston and Watkins, 1999), Monte Carlo Tree Search (MCTS) with Neural Networks (Paxton et al., 2017), and Convolutional Neural Networks (CNNs) (Lei et al., 2018) belong to this class. Out of all the ML-related frameworks, the most relevant to this work is RL, where Q-learning and policy gradient have been employed (Zhou et al., 2022). More specifically, applications involve RL with PRM (Francis et al., 2020), where noisy sensors and complicated models are considered as well as Deep Reinforcement Learning (DRL) for visual navigation (Devo et al., 2020), where exploration and target-following tasks are considered. Another impressive DRL result is presented in (Miki et al., 2022), where a quadruped platform is demonstrated to tackle challenging, real-world environments.

Finally, with regards to more modern treatments of MP, several promising approaches have arisen, such as Non-linear Model Predictive Control (Grandia et al., 2022) and locally reactive controllers (Mattamala et al., 2022). Be that as it may (even though in general MPC can be employed to solve kinematic problems, albeit with large computational resources to avoid local minima), the above impressive and sophisticated controllers differ from our approach, as we treat the *kinematic* path planning problem. Furthermore, the scope of our method is not limited in the extraction of a **single** path; our method provides a velocity field for the entirety of a robot’s physical workspace and can thus be employed as a higher lever planning module in combination with a low-level motion planner for robotic platforms. In that sense, the proposed method is more similar to conventional planners, e.g., RRT^⋆^, in the context of path extraction in a physical workspace. Within the same paradigm, Model Predictive Path Integral (MPPI) control (Mohamed et al., 2020; Williams et al., 2017 exhibits certain similarities to our approach and is demonstrated to work in complex case-studies. However, since the depicted results in the above works are limited to workspaces with numerous, but small and most importantly convex obstacles, these approaches might be susceptible to local minima (especially in the partial observability case), as long planning horizons (which might be necessary to escape local minima) might be computationally infeasible. Regardless, these methods also provide a single-path solution to MP, whereas the goal of the proposed method is to provide a solution for the entire workspace (w.r.t. a single goal position) in a one-shot manner, such that it can be used for many arbitrary navigation instances (w.r.t. the given final goal position).

Regarding the optimal MP problem, the aforementioned methods A^⋆^, D^⋆^, and Djikstra’s Algorithm minimize the solution’s path length with provable optimality in some cases, while RRT^⋆^ can be modified to encompass kinodynamic constraints and more complex cost function forms. Furthermore, the latter is also proven to provide asymptotic optimality (at the limit of infinite run-time). In practice, satisfactory sub-optimal solutions can be obtained, or if desired, post processing can improve existing paths (Geraerts and Overmars, 2007). Concerning non-linear dynamics, the authors (Li et al., 2016) propose a methodology that treats such cases, which is especially relevant to the herein proposed method. On the contrary, there has been limited work on optimal reactive navigation, with some works including optimal NFs for stochastic systems (Horowitz and Burdick, 2014), where either a complete solution necessitates solving a hard Partial Differential Equation (PDE), or semi-complete treatments implement parameter tuning (Vadakkepat et al., 2000; Amiryan and Jamzad, 2015). It is therefore clear that sampling/graph-based methods have been explored extensively for the optimal MP problem, whereas reactive/continuous methods leave a lot of issues unresolved.

### 1.2 Contributions

Given the preceding discussion, we propose a method that merges the advantages of continuous methods with the RL-induced optimality through a PI method for 2-dimensional, planar optimal motion planning. The proposed framework additionally extends optimality to a set of non-linear, first order dynamics. More specifically, our contributions are.

1. A Policy Iteration algorithm that provides successively improving Reactive Motion Planning Policies,
2. A projection controller, such that safety during navigation and asymptotic convergence to the goal position are guaranteed, and
3. A workspace decomposition scheme that improves the computational performance of the proposed method.

The “reactive optimal motion planning problem” concentrates on the extraction of a *reactive* input vector field, defined over a robot’s workspace, such that a Cost Function is minimized. The novelty in the first two points lies in the fact that the proposed method is *reactive* in the technical, control theoretic sense; that is, the policy (velocity input) is explicitly dependent on the state (position) of the robot (feedback). While this might also be the case for many modern RL methods (where the term reactive is rarely employed), our work is aligned with the control theoretic paradigm of treating the evolution of Ordinary Differential Equations (ODEs) (as in the flows of vector fields) over manifolds. This also demonstrates the main difference between the proposed scheme and MPC-based ones; the herein proposed offline optimization enables extracting optimal trajectories from anywhere within the workspace, w.r.t. a given final position, without the need for re-running the method.

Therefore, the proposed optimal control problem essentially consists of a functional analysis problem, and requires the solution of a hard, non-linear, PDE. The RL-scheme is employed appropriately to construct the optimal input without necessitating the solution of the aforementioned PDE. Instead, the cost function gradient is successively approximated through gathered data, and at the same time CT is employed to provide safety, convergence, and policy improvement guarantees. As it will become apparent in the sequel, full knowledge of the dynamics does not enable extracting the cost function *a priori*, therefore successive approximation is necessary. For more details, the reader is directed to (Abu-Khalaf M. and Lewis F. 2005).

### 1.3 Outline

The rest of the manuscript is organized as follows: In Section 2.1, we formulate the problem in the language of control theory. Subsequently in Section 2.2, we present the proposed method and provide some formal analysis that motivates our approach. In Section 2.3, we prove the asserted technical claims of the method and continue with providing an overview of the scheme, along with some practical details in Section 2.4. In Section 2.5, a scheme for ameliorating the computational characteristics of the proposed method is presented. Finally, we present rigorous simulation results in Section 3, with the relevant figures included in a separate section for readability, and conclude with a relevant discussion and envisioned future prospects in Section 4.1.

## 2 Materials and methods

### 2.1 Optimal motion planning as an optimal regulation (OR) problem

**FIGURE 1:**
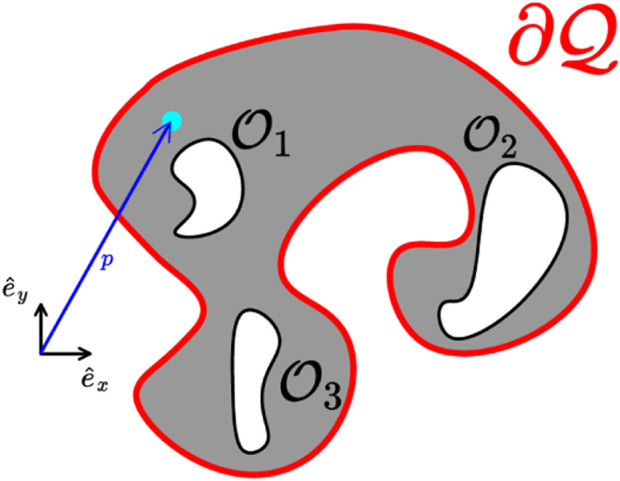
A workspace, whose outer boundary is depicted in a red line, and three obstacles depicted with black lines. A frame of reference along with an exemplary position (cyan disk), and the corresponding position vector (blue arrow) are also depicted.

Consider a point robot^1^ operating within a fully known, bounded (with external walls), and connected planar workspace $Q\subset R^{2}$ , with *M* inner distinct obstacles $O_{i}\subset R^{2},\;i=1,\ldots ,M$ . The free workspace is given by $W=Q-{⋃}_{i=1}^{M}O_{i}$ and its boundary is given by ∂W . An example of the aforementioned defined quantities is depicted in Figure 1. ^2^Consider also the desired, final position of the robot denoted by $p_{d}\in W-\partial W$ . The robot’s motion is dictated by the following, first-order system of non-linear ODEs:

$$\dot{p}=f\left(p\right)+u,\;p\left(0\right)=\bar{p},$$ (1)

where p∈W−∂W denotes the robot’s position, $f\left(p\right):W\mapsto R^{2}$ models non-linear first order dynamics, which may correspond to the robot’s non-linear dynamics or relevant interactions with its environment and is considered to be fully known, $u\left(t\right):\;R_{+}\mapsto R^{2}$ denotes the velocity command applied to the robot and $\bar{p}\in W-\partial W$ denotes the robot’s position at time *t* = 0 (initial position). In order to formulate the optimal control problem, we make the following assumptions.

1. *f* is a Lipschitz continuous function for all p∈W ,
2. *f* vanishes at the desired position, i.e., *p*_*d*_ is an equilibrium of system (1).

Assumption 1 is common for addressing control problems and roughly concerns the absence of singularities inside the workspace. Assumption 2 is stronger and might not hold for real-world applications, however it will be shown in the sequel that it can be relaxed by applying a fixed translation to the input signal, i.e., *u* = *u*′ − *f*(*p*_*d*_), to render *p*_*d*_ an equilibrium, where *u*′ denotes the new input command.

Remark 1 Form (1) is motivated by agents that operate within environments where, in addition to the velocity input, the external environmental interactions are modeled through a velocity field. For instances such as of an underwater vehicle operating in the presence of an external water flow (sea currents), the robot velocity depends on both the input, and the position-dependent flow (i.e., on the term f(p) in Eq. (1)) induced by the environment (Fossen, 1994). Since our work is limited to first order dynamics, the adopted model could be used as an approximation of the motion of a robotic vehicle in the presence of such an external fluid flow. Such an approach, although approximate, would still prove beneficial to address energy consumption, especially in slow-moving flows, if the underlying optimal velocity field is employed as a high-level velocity command to a low-level tracking controller. Tracking a straight line trajectory (the optimal path if fluid flow is neglected) towards a goal position would result in larger amounts of energy consumption compared to a trajectory incorporating fluid flow expressed in the adopted simplified velocity space. On the other hand, if one were to cancel the drift term f(p) and deal with a single integrator model then the solution would not be optimal (see (Freeman and Kokotovic 1996)), as the drift term may be advantageous in guiding the robot to the goal position (depending on the flow). Finally, the technical results provided in the sequel can easily be extended to systems of the form $\dot{p}=f\left(p\right)+g\left(p\right)u,\;p\in W-\partial W$ for cases where $g\left(p\right):W\mapsto R^{2\times 2}$ is a full-rank (thus invertible) matrix.

The **goal** of this work is to design a **reactive control input** (i.e., a vector field) $u\left(p\right):W\mapsto R^{2}$ such that the following infinite-horizon cost function is minimized:

$$V_{u}\left(\bar{p}\right)=\int_{0}^{\infty }Q\left(p_{u}\left(\tau ;\bar{p}\right);p_{d}\right)+R\left(u\left(\tau \right)\right)d\tau ,$$ (2)

where $p_{u}\left(t;\bar{p}\right):\;R_{+}\mapsto W$ denotes the trajectory that stems from integrating (1) while applying the control input *u*, starting (at time *t* = 0) from the initial position $p\left(0\right)=\bar{p}\in W-\partial W$ , and the index *u* indicates that the cost corresponds to the input signal *u*. Furthermore, we define the **state-related cost term** *Q* and the **input-related cost term** *R* respectively.

$$Q\left(p;p_{d}\right)=\alpha {\left\|p-p_{d}\right\|}^{2},$$ (3a)

$$R\left(u\right)=\beta {\left\|u\right\|}^{2},$$ (3b)

where *α*, *β* are positive real-valued weighting constants and ‖ ⋅‖ denotes the Euclidean norm. The metric (2) along with (3) essentially result in a cost function from optimal regulation theory (Kalman, 1960). The state-related term Eq. 3a) can be understood as minimizing the *settling time* of the system, i.e., the time until the robot reaches an area close to the goal, since it penalizes the robot for staying away from the goal position as time evolves. The input-related term Eq. 3b) is more straightforward, as it penalizes the control input’s Euclidean norm, which, when integrated, roughly translates to *actuator energy minimization*.

The cost function $V\left(\bar{p}\right):W-\partial W\mapsto R^{+}$ can be interpreted as describing the cost that is accumulated along a trajectory of system (1) starting from the initial position $\bar{p}$ . For the above cost to be well-defined (i.e., for the limit of the integral (2) to be converging to a finite value), the trajectory should be asymptotically converging to the goal position, with the latter consisting an equilibrium of (1). This is true only if Assumption (2) holds, as in any other case, a non-zero value for the state-related cost term would accumulate when integrated for infinite time, resulting in an infinite cost function value. Nevertheless in case $f\left(p_{d}\right)\neq \tilde{0}$ , we can redefine the cost-related term as:

$$R^{\prime }\left(u\right)=\beta {\left\|u+f\left(p_{d}\right)\right\|}^{2},$$ (4)

Which renders the limit of the integral (1) well-defined for asymptotically converging trajectories.

Finally, while the drift dynamics are assumed to be fully-known, we underline that the OR problem is still non-trivial; that is, the fact that neither the optimal cost function (2), nor the cost function gradient are readily available, renders the extraction of the optimal policy rather difficult, requiring the solution of a hard, non-linear PDE. In the sequel we bypass this limitation through the proposed RL-PI scheme.

### 2.2 Proposed method

In this section, the proposed PI scheme will be presented. First of all, we begin by defining the set of **admissible policies** which serves as a basis for designing a framework for a reactive solution with provable guarantees.

Definition 1 (***Admissible Policy***) *A navigation policy* $u\left(p\right):W\mapsto A\left(W\right)$ *, where* $A\left(W\right)$ *denotes the set of admissible policies, is defined as admissible with respect to the cost function* (2) *over the workspace* W *, if:*

1. *u* *is continuous on* W *,*
2. $f\left(p_{d}\right)+u\left(p_{d}\right)=\tilde{0}$ *,*
3. $u\left(p\right)$ *stabilizes* (1) *on* W *,*
4. $V_{u}\left(p\right)$ *is finite* ∀p∈W *and*
5. *the resulting trajectories of* (1) *under the control law* $u=u\left(p\right)$ *are safe, i.e., for any* $\bar{p}\in \left(W-\partial W\right)$ *it holds that* ^3^

$$\begin{array}{c} P_{u}\left(\bar{p}\right)⋂\partial W=\emptyset ,\text{where: }P_{u}\left(\bar{p}\right)=\underset{t\in \left[0,+\infty \right)}{⋃}p_{u}\left(t;\bar{p}\right). \end{array}$$

#### 2.2.1 Initial policy

In RL, the training process begins through the implementation of an initial, sub-optimal policy that serves as a starting point for the optimization algorithm. It is standard practice that the initial policy is acquired through its parametrization via an approximation structure and the initialization of its parameter vector. As will become apparent in the sequel, our policy is on the contrary **parameter-free**. However, a vector field that can be employed as a reactive^4^ and admissible initial policy is also required (see Def. 1). As discussed in Section 1.1, there exist a plethora of methodologies for providing provably correct reactive vector fields in the literature. In Rousseas et al. (2022), we have provided a method for obtaining such an initial policy, through an AHPF^5^:

$$v\left(p\right)=-\exp\left(\phi^{T}\left(p\right)w\right)\nabla \phi \left(p\right)w,$$ (5)

where $w≜{\left[w_{0},w_{1},\ldots ,w_{K}\right]}^{T}\in R^{K+1}$ are the respective weights of the structure, $\phi \left(p\right)≜{\left[\phi_{0}\left(p;p_{d}\right),\phi_{1}\left(p;p_{1}\right),\ldots ,\phi_{K}\left(p;p_{K}\right)\right]}^{T}:\;R^{2}\mapsto R^{K+1}$ is the regressor vector of **harmonic terms** $\phi_{i}\left(p;p_{i}\right)=\ln\left(\left\|p-p_{i}\right\|\right)$ and *K* + 1 is the overall number of harmonic terms. This field, when the weights of the structure are appropriately tuned, exhibits a single stable equilibrium at the desired position $p_{d}\in W-\partial W$ , while it furthermore points inwards at the boundary, thus providing safe navigation to the goal position. Such a field will henceforth be referred to as a *Navigation Field*. We direct the reader to (Rousseas et al., 2022) for details over the aforementioned method^6^. Through this velocity field, the initial policy, which will henceforth be denoted as:

$$u^{\left(0\right)}\left(p\right)=u^{\left(i=0\right)}\left(p\right)=-f\left(p\right)+v\left(p\right),$$ (6)

Renders system (1) almost Globally Asymptotically Stable (GAS). The term *“almost”* relates to the topology of the workspace and is discussed extensively in the following subsection.

#### 2.2.2 Impossibility of strictly global navigation in multiply connected workspaces

Prior to presenting the proposed scheme, it is necessary to discuss some properties of continuous vector fields over multiply connected manifolds (i.e., workspaces with internal obstacles). In their seminal work, Koditschek and Rimon (1990) proved how in multiply connected manifolds, no strictly globally attractive vector field can be defined. This limitation stems from saddle-points of the vector field, whose existence is guaranteed through the following proposition.

Proposition 1 (Corollary 2.3 in (Koditschek and Rimon 1990)). *Let* *v* *be a smooth nondegenerate vector field on the free space,* W−∂W *, with* *M* > 0 *obstacles, which is transverse on* ∂W *. Suppose that* *v* *has a unique attracting equilibrium point. Then each obstacle introduces at least one saddle point of* *v*.

We now employ the above proposition to show how limiting the vector field to the form (5), as introduced in Rousseas et al. (2022), presents the advantage of exhibiting as few saddle points as possible.

Proposition 2 *Let* $W=Q-{⋃}_{i=1}^{M}O_{i}$ *denote a Jordan* (*the boundary of* W *consists of disjointed Jordan curves*) *multiply connected workspace (manifold) embedded in the Euclidean plane* $\left(W\subset R^{2}\right)$ *, with* *M* *internal obstacles. A harmonic navigation field* (5)*, defined over* W *exhibits exactly* *M* *saddle points in* $int\left(W\right)$ .

Proof. *See* Supplementary Appendix S1 *.*

The above proposition essentially describes how AHPFs, owing to the minimum-maximum principle and the subsequent lack of any local maxima in the interior of a workspace^7^, induce only as many saddle points as the number of obstacles.

The restrictions on the existence of continuous reactive controllers in the presence of obstacles that are discussed in this section have recently been formalized and generalized through the notion of *Topological Perplexity* (TP) in Baryshnikov (2021). The effect of TP and the above propositions on our method is twofold: First of all, while Definition 1 can be employed in the context of simply connected manifolds (workspaces with no internal obstacles) and harmonic vector fields, this is not the case when internal obstacles are present. The hyperbolic equilibria (saddles), impart *stable manifolds* from which trajectories fail to reach the goal position. Additionally, any saddle of the Navigation Field will result in infinite cost for any point that starts *exactly* at the stable manifold of the former. This is proven in Proposition 3. Nevertheless, the aforementioned manifolds are of Lebesque measure one, therefore the probability of starting exactly on them (and thus the probability of the navigation failing) is zero. To formalize this notion, we adopt the definition of a stable manifold, *W* ^*st*^(*p*_*s*_) of the saddle point *p*_*s*_, *s* = 1, … , *M* from (Hsu, 2013)^8^:

$$\begin{array}{cc} W_{u}^{st}\left(p_{s}\right) & =\left\{\bar{p}\in W:p_{u}\left(t;\bar{p}\right)\text{is defined for }t\geq 0\right.\\ & \;\left.\text{and }\underset{t\mapsto \infty }{\lim}\left(p_{u}\left(t;\bar{p}\right)\right)=p_{s}\right\} \end{array}$$ (7)

where $p_{u}\left(t;\bar{p}\right)$ is the flow of (1) (i.e., trajectory) from the initial point $p\left(0\right)=\bar{p}\in W$ up to time *t* > 0.

Proposition 3 *Given the harmonic navigation Field* *v* *in* (5) *the cost function* (2)*, evaluated on trajectories of the system* $\dot{p}=v\left(p\right)$ *, obtains an infinite value for any starting points lying on the stable manifolds of the saddle points of* *v* ^9^.

Proof.*See* Supplementary Appendix S1 *.*

#### 2.2.3 Addressing multiply connected workspaces

The singularity that the cost function admits, and which was proven to exist in Proposition 3, yields technical and practical challenges for the application of the proposed PI scheme, as the approximation of functions with singularities is ill-posed. The proposed method is still applicable for obstacle-free workspaces, as the lack of internal equilibria is guaranteed through Proposition 2. We therefore propose applying our method in multiply connected workspaces through generating a transformed workspace $W^{\prime }$ , from the initial workspace W , where the obstacles are linked to the *outer* boundary ∂Q of the initial workspace through the addition of measure-two (of finite area) artificial-boundary regions, thus rendering the workspace simply connected. We underline that the simply-connected version of the workspace always exhibits a tree-like topology, as by definition, any closed loop in the connectivity graph implies the existence of obstacles. Hence, the construction of the transformed workspace that is described in the sequel always yields such a topology. The effects of the above process are.

1. A region of finite area is rendered inaccessible for the robot,
2. The topology and geometry of the artificial-boundary regions directly influence optimality, i.e., a poor choice of such regions can hinder the optimality of the final controller (upon convergence).

However.

1. Such an initial subdivision of a multiply-connected workspace to a simply-connected one is unavoidable (be it through one-dimensional sets instead of two-dimensional ones) due to the presence of saddles,
2. The newly simply connected workspace admits Admissible policies, as per Definition 1,
3. The cost on the boundaries of the resulting harmonic navigation field is finite,
4. The regions can be rendered arbitrarily small, as long as they remain of measure-two.

**FIGURE 2:**
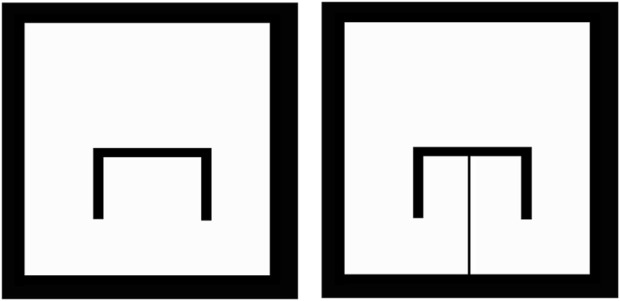
The Original Workspace, containing a *π*-shaped obstacle (left) with its Simply-Connected, Transformed Version (right). The obstacle is connected to the boundary through a slim region. The free part of the workspace is depicted in white, while the occupied part of the workspace is depicted in black.

The aforementioned step is thus a well-motivated and necessary one in order to formulate a PI scheme without sacrificing mathematical exactness. Negating the need for such a transformation is furthermore a promising future research direction that we intend to pursue. An example of this process is presented for a simple workspace in Figure 2. It can be shown that the herein depicted choice for linking the obstacle to the outer boundary might not be optimal for several choices of desired final robot positions (e.g., for *p*_*d*_ positioned “within” the convex region defined by the *π*-shaped obstacle).

**FIGURE 3:**
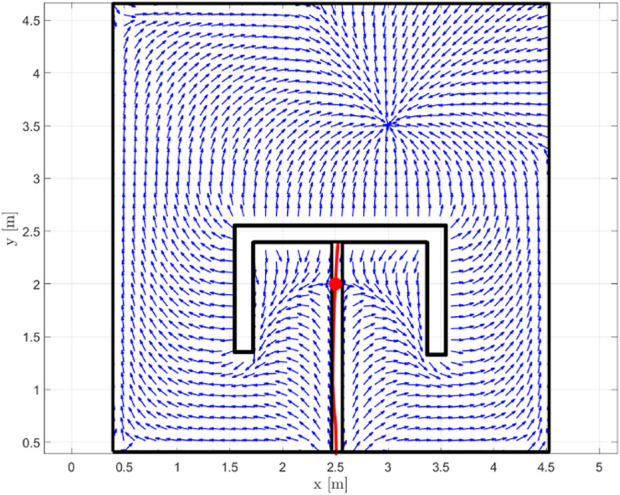
An AHPF for the multiply-connected obstacle, along with the saddle (red disk), the stable manifold (red, continuous line) and its bounding box (black) that is employed to render the workspace simply connected.

In order to obtain a transformed workspace such as the one in Figure 2, we leverage Proposition 2, along with the method in Rousseas et al. (2022). Briefly, we initially obtain an AHPF through the method in Rousseas et al. (2022), for the **multiply connected** workspace. According to Proposition 2, the vector field (5) admits *M* saddles, each of which further consist of two (one-dimensional) manifolds, a stable and an unstable one (Hsu, 2013). It can be easily shown by contradiction that, for a safe AHPF with a sink at the workspace’s interior, the unstable manifold of a saddle connects the latter to the goal position, while the stable manifold connects the saddle to the boundary^10^. Consecutively, the saddles, along with their stable manifolds can be computed numerically, which essentially provides a set of curves that link the obstacles to themselves and/or the boundary. In order to turn the one-dimensional manifolds into the two-dimensional ones that are necessary for the method, simple algorithms can be implemented, such as a bounding box one. The respective field, along with the stable manifold and its bounding box, are depicted in Figure 3.

#### 2.2.4 Policy iteration scheme

In this section, the proposed PI scheme is presented. In PI methods, the robot executes a sub-optimal policy and computes the related cost function (2), through a computationally expensive process. Subsequently, the acquired information is employed in order to ameliorate the policy w.r.t. to the cost function. This process is repeated until the cost function converges. While the computational cost of PI methods is significant, owing to the extensive cost data gathering, the convergence rate is fast (Brunton and Kutz, 2021), as in practice only a small number of training “epochs” (i.e., iterations) are needed. As the TP-related issues were discussed in Subsections 2.2.2 and 2.2.3, henceforth we treat the case where the initial policy exhibits no internal critical points, and we employ the symbol $W^{\prime }$ to denote the transformed, simply-connected (i.e., obstacle-free) version of the workspace where appropriate, as discussed in Subsection 2.2.3. In the following subsections, we begin by extracting the optimal policy, and subsequently providing a theory-inspired efficient solution to the cost function data gathering step.

#### 2.2.5 Optimal policy

To extract the optimal policy, note that for any GAS policy *u* defined over $W^{\prime }$ (even if it is not safe *per se*), the following differential form of (2) can be formulated:

$$\begin{array}{ll} {\left(\nabla_{p}V_{u}\right)}^{T}\dot{p} & =-r\left(p,u\right)\Leftrightarrow {\left(\nabla_{p}V_{u}\right)}^{T}\left(f\left(p\right)+u\right)\\ & =-\alpha {\left\|p-p_{d}\right\|}^{2}-\beta {\left\|u\right\|}^{2}, \end{array}$$ (8)

where the term *r* is defined as:

$$r\left(p,u\right)=Q\left(p;p_{d}\right)+R\left(u\right).$$ (9)

The above equation along with the **terminal condition**

$$V_{u}\left(p_{d}\right)=0,$$ (10)

Define a Lyapunov-like PDE. In order to minimize (2) the following Hamiltonian is constructed:

$$H\left(p,u;\nabla_{p}V\right)={\left(\nabla_{p}V_{u}\right)}^{T}\left(f\left(p\right)+u\right)+r\left(p,u\right).$$ (11)

Hence, the optimal cost function *V* ^⋆^ satisfies the Hamilton-Jacobi-Bellman (HJB) equation:

$$\underset{u}{\min}\left\{H\left(p,u;\nabla_{p}V^{⋆}\right)\right\}=0,$$ (12)

Which through applying the stationary condition on (11) results in the optimal policy:

$$\begin{array}{c} u^{⋆}=\underset{u}{\mathrm{arg min}}\left\{H\left(p,u;\nabla_{p}V^{⋆}\right)\right\}\Rightarrow u^{⋆}\left(p\right)=-\frac{1}{2\beta }\nabla_{p}V^{⋆}\left(p\right), \end{array}$$ (13)

where *V* ^⋆^ denotes the optimal cost function. Additionally, in the context of successive approximation^11^, as presented by Abu-Khalaf and Lewis, 2005b), given a GAS policy *u* ^(*i*)^, where the index *i* denotes the *i*th step of the successive process, the associated cost:

$$V^{\left(i\right)}≜V_{u^{\left(i\right)}},$$ (14)

Resulting from applying *u* ^(*i*)^ to (1) can be employed through the feedback control law:

$$u^{\left(i+1\right)}=-\frac{1}{2\beta }\nabla_{p}V^{\left(i\right)}\left(p\right)$$ (15)

And can be shown to result in global asymptotic stability for (1), and at the same time improving the cost in the entire state-space. However, through this formulation safety is not guaranteed, as throughout the preceding analysis the condition that the policy is admissible, and more specifically condition 5 of Def. 1, was never imposed. Indeed, while the extracted policy (13) is GAS for systems (1) according to Beard et al., 1995) and Abu-Khalaf and Lewis, 2005a), it is not necessarily safe. In order to obtain an optimal **safe** policy, safety needs to be incorporated in the control design.

#### 2.2.6 Admissible and cost improving policy

With the goals of preserving global asymptotic stability and control improvement as described in Abu-Khalaf and Lewis. (2005b) for the PI scheme (15), as well as imbuing system (1) under the feedback control law *u* ^(*i*+1)^ with safety, we propose the following policy:

$$u^{\left(i+1\right)}\left(p\right)=\left\{\begin{array}{cc} -\frac{1}{2\beta }\nabla_{p}V^{\left(i\right)}\left(p\right),\; & p\notin S_{a}\\ -\frac{1}{2\beta }\nabla_{p}V^{\left(i\right)}\left(p\right)+u_{C}^{\left(i\right)}\left(p\right),\; & p\in S_{a} \end{array}\right.,$$ (16)

where the set $S_{a}$ is defined as:

$$S_{a}=\left\{p\in W^{\prime }\;|\;d\left(p\right)\leq a\right\},$$ (17)

With $a\in R_{+}$ being a tuning parameter and where the distance function $d:W^{\prime }\mapsto R_{+}$ is given by

$$d\left(p\right)=\underset{z\in \partial W^{\prime }}{\min}\left\{\left\|p-z\right\|\right\},$$ (18)

**FIGURE 4:**
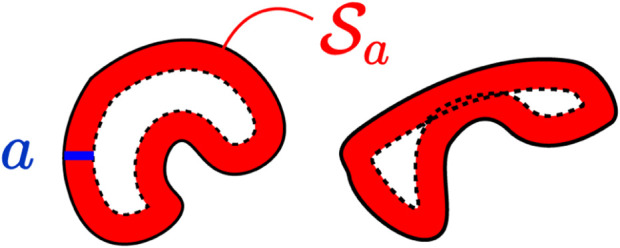
Two indicative workspaces (black) and the safety-check set $S_{a}$ depicted by the red-shaded area. The distance *a* is also depicted in blue. Correct choice of *a* (left, no overlap), incorrect choice of *a* (right, overlap).

And which corresponds to the distance of any point p∈W to the workspace boundary. The set (17) defines an area of distance $a\in R_{+}$ ^12^ around the workspace’s boundary. An example of the set $S_{a}$ is depicted in Figure 4 for an exemplary workspace. Outside the set $S_{a}$ the control law assumes the optimal value that results from the stationary condition (13). Inside this area, a correction term is added to the control law:

$$\begin{array}{ll} u_{C}^{\left(i\right)} & =\frac{b_{a}\left(d\left(p\right)\right)}{2\beta }\nabla_{p}V^{\left(i\right)}\left(p\right)-b_{a}\left(d\left(p\right)\right)f\left(p\right)\\ & \;-b_{a}\left(d\left(p\right)\right)\frac{{\left(\nabla_{p}V^{\left(i\right)}\right)}^{T}\left(f\left(p\right)+u^{\left(i\right)}\right)}{2\beta {\left\|f\left(p\right)+u^{\left(i\right)}\right\|}^{2}}\left(f\left(p\right)+u^{\left(i\right)}\right), \end{array}$$ (19)

where the third term is essentially the projection of the cost function gradient on the robot’s dynamics under the previous input, i.e., ${pr}_{\left({\dot{p}}^{\left(i\right)}\right)}\left(\nabla_{p}V^{\left(i\right)}\right)$ , with ${\dot{p}}^{\left(i\right)}=f\left(p\right)+u^{\left(i\right)}$ . The *bump function* $b_{a}\left(x\right):\;R_{+}\mapsto \left[0,1\right]$ is defined as

$$b_{a}\left(x\right)=\left\{\begin{array}{cc} \exp\left(-{\left(\frac{x}{x-a}\right)}^{2}\right),\; & x\leq a\\ 0,\; & x>a \end{array}\right.,$$ (20)

which is a continuously differentiable (but not analytic) function that varies from 1 to 0 over a distance *a*. In order for the function:

$$b_{a}\;◦\;d\left(p\right):\;W^{\prime }\mapsto \left[0,1\right]$$ (21)

In (19) to be properly-defined, the parameter *a* should be set as small as possible, such that: i) there is no ambiguity over which part of the boundary is considered in the distance function (18)—the red regions of Figure 4 should not overlap—and ii) the goal position does not lie within the area $S_{a}$ .

The control law (16) along with (19) can be understood as follows: away from the workspace boundary (outside the set $S_{a}$ ), the control law assumes the value of the negated gradient of the cost function that results from applying the previous policy. This is the best implementable policy according to the information encoded in the cost function of the previous policy (and it may therefore be characterized as a greedy policy). As the robot approaches the boundary, it enters $S_{a}$ and the bump function smoothly introduces the extra terms in (19). The first two terms smoothly nullify the drift dynamics and the input applied outside $S_{a}$ , respectively. The third term smoothly projects the negated gradient of the cost function *V* ^(*i*)^ onto the previous dynamics *f*(*p*) + *u* ^(*i*)^. While the above projection controller is similar to the barrier function formulation, it differs critically in that the projection is employed w.r.t. the previous policy. This (along with the initial admissible policy extraction) results in a **constructively safe** controller, whereas in case of barrier functions, a suitable barrier function that renders the trajectories of the system safe needs to be discovered first. In the following section we prove how the proposed controller (16) satisfies the asserted properties.

Remark 2 *In order to treat the dynamics discussed in Remark* 1 *with the additional input multiplier* *g*(*p*)*, the optimal input* (13)*—and its equivalent in* (16)*—should be altered as follows:*

$$u^{\left(i+1\right)}=-\frac{1}{2\beta }g^{\text{T}}\left(p\right)\nabla_{p}V^{\left(i\right)}\left(p\right).$$ (22)

*Additionally, the correction term can be enhanced through pre-multiplying with* *g* ^−1^(*p*)*—as a reminder, this concerns the case where* *g*(*p*) *is invertible. Conversely, the technical results in the sequel can be easily extended, however the relevant proofs are omitted for clarity of exposition and readability.*

### 2.3 Technical results

In this section, the asserted technical claims are proven, namely, safety, global asymptotic convergence, and control improvement.

#### 2.3.1 Existence and safety

The feasibility of the correction term (19) necessitates the existence of the projection at the *i*th iteration^13^, i.e.,:

$${\left(\nabla_{p}V^{\left(i\right)}\right)}^{T}\left(f\left(p\right)+u^{\left(i\right)}\right)\neq 0.$$ (23)

To see this, note that through (8) the projection is equal to

$${\left(\nabla_{p}V^{\left(i\right)}\right)}^{T}\left(f\left(p\right)+u^{\left(i\right)}\right)=-\alpha {\left\|p-p_{d}\right\|}^{2}-\beta {\left\|u^{\left(i\right)}\right\|}^{2},$$ (24)

Which implies that indeed (23) holds, as the Right-Hand Side (RHS) of (24) is strictly negative $\forall p\in W^{\prime }-\left\{p_{d}\right\}$ . Since *a* is chosen such that $p_{d}\notin S_{a}$ (see the previous section), the last term in (19) is a well-defined, smooth projection for any $p\in W^{\prime }$ . To prove global asymptotic stability, we first of all show that *u* ^(*i*+1)^ is bounded in Lemma 1 and subsequently prove that the proposed policy is safe in Lemma 2.

Lemma 1 (**Boundedness of the input**). *Given an admissible policy* *u* ^(*i*)^ *, the policy* *u* ^(*i*+1)^ *is bounded* $\forall p\in W^{\prime }$ .

Proof. It suffices to prove that each term in the sum (16) is bounded. Owing to admissibility of *u* ^(*i*)^, ∇_*p*_ *V* ^(*i*)^ is bounded. Therefore, owing to boundedness of *b*_*a*_(*d*) the only non-trivial term is ${pr}_{\left({\dot{p}}^{\left(i\right)}\right)}\left(\nabla_{p}V^{\left(i\right)}\right)$ . Note that, from (8):

$$\begin{array}{cc} \left\|{pr}_{\left({\dot{p}}^{\left(i\right)}\right)}\left(\nabla_{p}V^{\left(i\right)}\right)\right\| & =\left\|\frac{-r\left(p,u^{\left(i\right)}\right)\left(f\left(p\right)+u^{\left(i\right)}\right)}{2\beta {\left\|f\left(p\right)+u^{\left(i\right)}\right\|}^{2}}\right\|\\ & =\frac{r\left(p,u^{\left(i\right)}\right)\left\|f\left(p\right)+u^{\left(i\right)}\right\|}{2\beta {\left\|f\left(p\right)+u^{\left(i\right)}\right\|}^{2}}\\ & =\frac{\alpha {\left\|p-p_{d}\right\|}^{2}+\beta {\left\|u^{\left(i\right)}\right\|}^{2}}{2\beta \|f\left(p\right)+u^{\left(i\right)}\|}, \end{array}$$

Which, owing to the admissibility of *u* ^(*i*)^ is bounded *∀p* ≠ *p*_*d*_, owing to the denominator being non-zero (as this would imply a local minimum inside the workspace). For *p* → *p*_*d*_, note that the denominator tends to 0. However, since the term ‖*u* ^(*i*)^‖^2^ converges to zero faster than ‖*u* ^(*i*)^‖, the fraction is well-defined and also converges to 0 for *p* → *p*_*d*_, which concludes the proof.

Lemma 2 (**Safety of the input**). *The control law* (16) *applied to System* (1) *results in safe trajectories according to Definition* 1 *for any initial point* $\bar{p}\in W^{\prime }$ *, if the previous policy* *u* ^(*i*)^ *is also safe* $\forall \bar{p}\in W^{\prime }$ .

Proof. In order to prove safety, note that owing to the first-order dynamics (1), the safety condition of Definition 1 is equivalent to the velocity of the robot pointing inwards at the boundary, i.e.,

$${\dot{p}}^{T}\left(z\right)n\left(z\right)>0,\;z\in \partial W,$$ (25)

where *n*(*z*) is the inwards-pointing vector that is normal to the boundary at the point $z\in \partial W^{\prime }$ . Notice also that under the control law (16) and the fact that for any z∈∂W , *b*_*a*_(*d*(*z*)) = 1, Eq. 16, 19 and 20, yields:

$$\begin{array}{c} {\dot{p}}^{T}\left(z\right)n\left(z\right)=-\frac{{\left(\nabla_{p}V^{\left(i\right)}\right)}^{T}\left(f\left(z\right)+u^{\left(i\right)}\right)}{2\beta {\left\|f\left(z\right)+u^{\left(i\right)}\right\|}^{2}}{\left(f\left(z\right)+u^{\left(i\right)}\right)}^{T}n\left(z\right). \end{array}$$

Hence, if the *i*th policy is safe, then ${\left(f\left(z\right)+u^{\left(i\right)}\right)}^{T}n\left(z\right)>0$ , and since through (8) ${\left(\nabla_{p}V^{\left(i\right)}\right)}^{T}\left(f\left(z\right)+u^{\left(i\right)}\left(z\right)\right)<0,\;\forall z\in \partial W^{\prime }$ , we conclude that ${\dot{p}}^{T}\left(z\right)n\left(z\right)>0$ , which concludes the proof.

#### 2.3.2 Main technical results

In this subsection, the main technical results are presented in Theorem 1. We prove how (16) results in Global Asymptotic Stability of System (1) through common Lyapunov arguments. A sketch of the proof begins by following the analysis by Abu-Khalaf and Lewis, (2005b) which coincides with our case for any $p\notin S_{a}$ . Subsequently, owing to the smoothness of the application of the correction term (19), we demonstrate how the resulting extra term does not hinder the Lyapunov argument.

Additionally, we explore the improvement of the cost function given the sequence of admissible policies (16). Abu-Khalaf and Lewis, (2005b) prove how a successive approximation scheme for nonlinear systems results in policy improvement. However, the workspace boundary in the context of motion planning alters the technical results significantly. We begin by examining the case for the single integrator, in which case the policies are provably improved w.r.t. to the cost function. Subsequently, we show that owing to the general form of the nonlinear term (1) the improvement of the cost function in that case can not be guaranteed for the entire workspace (although it is also not explicitly prohibited). Finally, a brief discussion over the case with drift dynamics is provided.

Theorem 1 (**Global Asymptotic Stability and Control Improvement**). *Given an* ***admissible*** (*see Definition* 1) *policy* *u* ^(*i*)^ *that results in Global Asymptotic Stability of System* (1) *and the associated cost function* *V* ^(*i*)^ *in* (2)*, the policy* (16) *also results in Global Asymptotic Stability of System* (1)*, which also renders* (16) *admissible. Furthermore, given the admissible policy* $u^{\left(i\right)}\in A\left(W^{\prime }\right)$ *, the policy* (16) *applied to System* (1) *with zero drift dynamics, i.e.,* $f\left(p\right)=\tilde{0},\forall p\in W^{\prime }$ *, results in improvement of the cost function* (2)*, i.e.,* *V* ^⋆^ ≤ *V* ^(*i*+1)^ ≤ *V* ^(*i*)^.

Proof. *See* Supplementary Appendix S1 *.*

In order to examine the case where $f\left(p\right)\neq \tilde{0}$ , the term *B*″ (from the proof of Theorem 1) becomes:

$$\begin{array}{ll} B^{\prime \prime } & =b\left(d\right)\left[\left(1-b\left(d\right)\right)\frac{\|\nabla_{p}V{\|}^{2}}{4\beta^{2}}+\frac{b\left(d\right)-1}{\beta }f^{T}\nabla_{p}V\right.\\ & \;-\frac{\left(1-b\left(d\right)\right)r^{2}\left(p,u\right)}{4\beta^{2}\|f+u{\|}^{2}}\left.-f^{T}\left(f+u\right)\left(1-\frac{b\left(d\right)r\left(p,u\right)}{2\beta {\left\|f+u\right\|}^{2}}\right)\right] \end{array}$$

Which yields:

$${\bar{B}}^{\prime \prime }\left(\left\|f\right\|\right)≜\bar{c}+\left(\bar{a}-\bar{b}\right)\|f\|-\|f{\|}^{2}+\frac{\bar{d}\|f{\|}^{2}+2\bar{d}\bar{b}\|f\|-\bar{g}}{\|f{\|}^{2}+2\bar{b}\|f\|+\bar{e}},$$

where the parameters in the above equation are omitted for brevity. Therefore, also noting that ${\bar{B}}^{\prime \prime }$ consists of a rational function and

$$\begin{array}{cc} {\bar{B}}^{\prime \prime }\left(0\right) & =\bar{c}-\frac{\bar{g}}{\bar{e}}=\frac{b\left(d\right)\left(1-b\left(d\right)\right)}{4\beta^{2}}\left(\|\nabla_{p}V{\|}^{2}-\frac{r^{2}}{\|u{\|}^{2}}\right)\\ & =\frac{b\left(d\right)\left(1-b\left(d\right)\right)}{4\beta^{2}}\|\nabla_{p}V{\|}^{2}\left(1-\cos\left(\theta \right)\right)>0, \end{array}$$

we conclude that:

$$\exists \bar{\left\|f\right\|}\in R|\forall f:\|f\|\leq \bar{\left\|f\right\|}\Rightarrow {\bar{B}}^{\prime \prime }\left(\left\|f\right\|\right)\geq 0.$$ (26)

For drift dynamics *f*(*p*) that obey (26), the successive application of the proposed policy (16) results in decreasing the cost function. Nonetheless, (26) is not constructive and we can not determine control improvement *a-priori*. Nevertheless, lack of control improvement over the **whole** workspace is the price for ensuring safety and convergence. Furthermore, control improvement is **guaranteed** for trajectories that do not cross $S_{a}$ , as through the preceding analysis it can be shown that *B*″ = 0 (note that all the terms are multiplied by *b*(*d*), which is zero for the aforementioned set of points). Additionally, note the dependence on the cost-related weighting parameter *β*. As the latter increases (which translates to demanding that the method reduces the applied input), the control improvement aspect of the algorithm deteriorates, which is intuitively expected if no bounds on the drift dynamics *f*(*p*) are imposed. We conclude that the lack of guarantees of control improvement is unavoidable in this case, as the safety correction term is explicitly dependent on the dynamics. We nevertheless demonstrate the ability of the proposed method to improve the cost function even in the presence of such non-linearities in Section 3.

Remark 3 (Global Optimality). In the preceding discussion, it was proven in Theorem 1 that the PI scheme provides a sequence of cost functions of decreasing level sets in the case for the single integrator dynamics, and under (26) for the non-linear case. However, this does not imply that the cost function upon convergence is the globally optimal one, as the sequence might converge to a different (higher) value for some points. Wang and Saridis (1992) have proven that, in a case where the input sequence is given by (15), the cost function upon convergence is nearly globally optimal (in the asymptotic sense)—see Theorem 5 in Wang and Saridis (1992). This holds true for our method as well, for the points where $u_{C}^{\left(i\right)}\left(t\right)=\tilde{0},\;\forall t\in \left[0,\infty \right]$ . However, in the case of multiply connected workspaces, this holds only for the transformed, simply-connected version of the latter; thus the solution depends heavily on the transformation from multiply to simply connected workspace. Therefore, strict global optimality is not attained by our method due to both aspects. In future works, we aim at addressing both aspects, i.e., providing the provably (nearly) globally optimal safe policy for multiply connected workspaces.

#### 2.3.3 Cost function computation - Data gathering

The proposed policy (16)–(19) requires the gradient of the cost function and full knowledge of the dynamics. While in RL algorithms unknown dynamics are usually treated, this work is limited to known dynamics, so as not to sacrifice provable guarantees. Nevertheless, treating unknown dynamics is an interesting and well-motivated direction that we intend to explore in the future. Additionally, in contrast to modern RL, where in many cases a Q-function is needed to map the state and policy to the cost, herein we only need to employ the gradient of the cost, as the mapping between the cost function and the optimal policy (w.r.t. the aforementioned instance of the cost function) is available through the HJB Equation 13. Thus, the PI scheme introduced herein suffices to improve upon the previous policies, as long as a representation for the cost function (or its gradient) is available.

In order to compute the cost function, we employ existing theoretical tools from PDE theory. We remind the reader that the relevant PDE consists of Equation 8 with the terminal condition (10), which is explicitly written in the semi-linear form:

$$\begin{array}{ll} & \frac{\partial V_{u}}{\partial x}\left(f_{x}\left(x,y\right)+u_{x}\left(x,y\right)\right)+\frac{\partial V_{u}}{\partial y}\left(f_{y}\left(x,y\right)+u_{y}\left(x,y\right)\right)\\ & \;=-r\left({\left[x,y\right]}^{T},u\left({\left[x,y\right]}^{T}\right)\right),V_{u}\left(p_{d}\right)=0, \end{array}$$ (27)

where $p={\left[x,y\right]}^{T},u=\left[u_{x}\left(x,y\right),u_{y}\left(x,y\right)\right]:\;W^{\prime }-\partial W^{\prime }\mapsto R^{2},f=\left[f_{x}\left(x,y\right),f_{y}\left(x,y\right)\right]:\;W^{\prime }-\partial W^{\prime }\mapsto R^{2}$ . In this subsection, we employ the explicit definition of the related fields for clarity (in place of the vector-matrix notation employed in the rest of the manuscript). Such PDEs can be solved explicitly via the method of characteristics (Nandakumaran and Datti, 2020). In summary, the 2-dimensional PDE is transformed into a system of 3 ordinary differential equations:

$$\frac{dx}{f_{x}\left(x,y\right)+u_{x}\left(x,y\right)}=\frac{dy}{f_{y}\left(x,y\right)+u_{y}\left(x,y\right)}=\frac{dV_{u}}{-r}.$$ (28)

In order to acquire a solution, the first step is to solve for the characteristic curve of the PDE by solving the first pair of ODEs, namely,

$$\begin{array}{c} \frac{dx}{f_{x}\left(x,y\right)+u_{x}\left(x,y\right)}=\frac{dy}{f_{y}\left(x,y\right)+u_{y}\left(x,y\right)}, \end{array}$$ (29)

where a characteristic line of the form *C* = *g*(*x*, *y*) will be obtained. However, note that in (28), the following:

$$\frac{dx}{f_{x}\left(x,y\right)+u_{x}\left(x,y\right)}=\frac{dy}{f_{y}\left(x,y\right)+u_{y}\left(x,y\right)}=dt,$$ (30)

**FIGURE 5:**
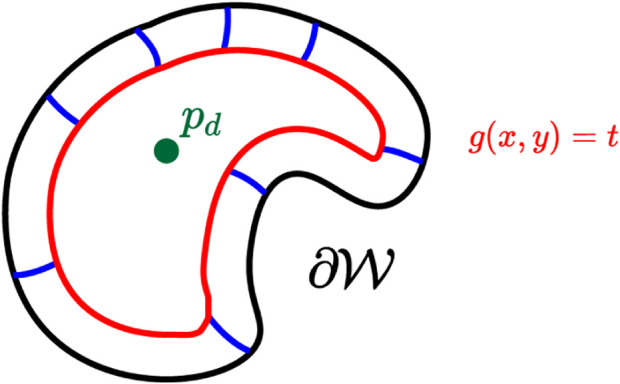
The characteristic solution *g*(*x*, *y*) = *t* (red line). The boundary (black line), the desired position (green disk). Trajectories are also depicted in blue lines linking the boundary to the characteristic.

Implies that the characteristic lines are the *isochronous curves* resulting from implementing the trajectories of (1), which are of the form *g*(*x*, *y*) = *t*, *t* ∈ [0, *∞*), an example of which is depicted in Figure 5. Note also that (30) admits the solution:

$$\begin{array}{c} x_{u}\left(t\right)=x\left(0\right)+\int_{0}^{t}\left(f_{x}\left(x,y\right)+u_{x}\left(x,y\right)\right)d\tau \text{, }\\ y_{u}\left(t\right)=y\left(0\right)+\int_{0}^{t}\left(f_{y}\left(x,y\right)+u_{y}\left(x,y\right)\right)d\tau , \end{array}$$ (31)

which is related to the preceding sections’ notation through the trajectory $p_{u}\left(t;{\left[x\left(0\right),y\left(0\right)\right]}^{T}\right)={\left[x_{u}\left(t\right),y_{u}\left(t\right)\right]}^{T}$ .

Finally, the solution of the remaining ODE for the cost function can be obtained:

$$\begin{array}{ll} \frac{dV_{u}}{-r} & =dt\Rightarrow V_{u}\left(x\left(t\right),y\left(t\right)\right)\\ & =\int_{0}^{t}\left(-r\left({\left[x\left(\tau \right),y\left(\tau \right)\right]}^{T},u\left({\left[x\left(\tau \right),y\left(\tau \right)\right]}^{T}\right)\right)\right)d\tau +C, \end{array}$$ (32)

where the constant C∈R is given through the terminal condition:

$$\begin{array}{ll} & \underset{t\to \infty }{\lim}\left[V_{u}\left(x\left(t\right),y\left(t\right)\right)\right]=V_{u}\left(p_{d}\right)=0\\ & \;\Rightarrow C=\int_{0}^{\infty }\left(r\left({\left[x\left(\tau \right),y\left(\tau \right)\right]}^{T},u\left({\left[x\left(\tau \right),y\left(\tau \right)\right]}^{T}\right)\right)\right)d\tau , \end{array}$$ (33)

Hence (33) is equivalent to evaluating the cost function for any point $\bar{p}\in W^{\prime }-\partial W^{\prime }$ . This formulation results to some ambiguity w.r.t. the starting time instant. This is implicitly defined in the initial conditions for the two state Eq. 31. In the following lemma, we prove how (33) is equivalent to (2), as the trajectories, starting from the boundary of the workspace cover the entire workspace.

Lemma 3 (**Full Coverage of the Workspace**). *The trajectories* (31) *of System* (1) *cover the entire workspace if every initial condition lying on the boundary of the workspace* $\bar{p}\in \partial W^{\prime }$ *is considered and for time* *t* ∈ [0, *∞*)*, i.e., for any* $p\in W^{\prime }$ *, there exists a unique tuple* $\left\{\bar{p},\bar{T}\right\}\in \partial W^{\prime }\times \left(0,\infty \right)$ *such that*

$$p=H\left(\bar{p},\bar{T}\right)≜\bar{p}+\int_{0}^{\bar{T}}\left(f+u\right)d\tau ,$$ (34)

*where* $H\left(\bar{p},\bar{T}\right)$ *is a homeomorphism.*

Proof. Let $p\in W^{\prime }$ . Propagating the dynamics (1) backwards for time *T* yields:

$$\begin{array}{cc} s & =p+\int_{0}^{T}\left(-f-u\right)d\tau =p-\int_{0}^{T}\left(f+u\right)d\tau \Leftrightarrow \\ p & =s+\int_{0}^{T}\left(f+u\right)d\tau , \end{array}$$ (35)

Which shows that there exist some $\left\{\bar{p},\bar{T}\right\}=\left\{s,T\right\}\in \partial W^{\prime }\times \left(0,\infty \right)$ . To complete the proof it suffices to show that $\left\{s,T\right\}\in \partial W^{\prime }\times \left(0,\infty \right)$ are unique for any $p\in W^{\prime }$ . This however is trivial. First of all, the solution of the system of ODEs (1) is unique (Khalil, 2014), owing to the fact that the dynamics along with the policy (16) are Lipschitz continuous $\forall p\in W^{\prime }$ . Finally, since the set $W^{\prime }$ is forward invariant, owing to admissibility of the policies, the complement $R^{2}-W^{\prime }$ is forward invariant for the backwards-propagated dynamics (35), yielding a single intersection point of the respective trajectory with $\partial W^{\prime }$ . Having established continuity and invertibility, we have essentially also proven that $H\left(\bar{p},\bar{T}\right)$ is a homeomorphism for $t\in \left[0,\infty \right)$ which concludes the proof.

#### 2.3.4 Reduction of dimensionality of cost function computation

The above lemma, besides proving equivalence of (33) to (2), for any position inside the workspace, additionally provides a way to reduce the dimensionality of the cost function computation. Normally, to obtain cost function values for any point inside the workspace, the integration of (33) for **every** such point is necessary. Even if the respective **two-dimensional** planar workspace was sampled, the complexity would prove intractable as the workspace size grows. However, Lemma 3 shows that it is sufficient to sample over the **one-dimensional** Jordan curve that forms the boundary of the workspace. This significantly reduces both the complexity and the scaling of the cost function computation. Starting from a discrete set of initial positions $p_{k,1}\in \partial W^{\prime },\;k=1,\ldots ,K$ , and taking $N_{k}\in N$ samples $p_{k,n}\in W^{\prime },\;n=1,\ldots ,N_{k}$ over the *k*th trajectory, $\bar{N}=\sum_{k=1}^{K}N_{k}$ samples for the cost function can be acquired from integrating (33) *K*-times. Note that (31) and 33 consist of a system of ODEs.

$$\frac{d}{dt}\left[\begin{array}{c} x\\ y\\ v_{u} \end{array}\right]=\left[\begin{array}{c} f_{x}\left(x,y\right)+u_{x}\left(x,y\right)\\ f_{y}\left(x,y\right)+u_{y}\left(x,y\right)\\ r\left({\left[x,y\right]}^{T},u\left({\left[x,y\right]}^{T}\right)\right) \end{array}\right],$$ (36)

With the initial conditions *x*(0), *y*(0), *v*_*u*_(0) = 0 whose solution yields the cost function (2), evaluated over points that lie on the computed trajectories of System (1):

$$V_{u}\left(x\left(t\right),y\left(t\right)\right)=\underset{t\to \infty }{\lim}\left(v_{u}\left(t\right)\right)-v_{u}\left(t\right),$$ (37)

where a dummy function *v*_*u*_ was employed to compute the cost function. Eq. 37 is essentially identical to (33), evaluated over the trajectories of System (1). In summary, the data gathered over the trajectories through the solution of (27) consist of tuples of the robot’s position, along with the corresponding cost values, i.e., $\left\{x_{i},y_{i},V_{i}\right\},\;{\left[x_{i},y_{i}\right]}^{\text{T}}\subset W,\;V_{i}\in R_{+},\;i\in \left\{1,\ldots ,\bar{N}\right\}$ , where $\bar{N}=\sum_{k=1}^{K}N_{k}$ is the total number of samples over all the computed trajectories and $N_{k}\in N$ is the number of points sampled over the *k*th trajectory.

Note that the above process yields the value of the cost function of an admissible policy at a collection of collocation points. However, this collection cannot be readily employed to extract a policy, as the **gradient** of the cost function is explicitly required for providing a control input. While numerical differentiation could provide a discrete form of the latter, in the sequel we propose a method to provide a continuous representation of the cost function gradient through DNNs. This approach is similar in scope to (Abu-Khalaf and Lewis, 2005a), where the optimal cost function is iteratively approximated.

### 2.4 The proposed PI scheme

In this section, the preceding elements are combined in a complete framework in the form of an algorithm. We also provide details regarding the implementation of our method in the following subsections.

#### 2.4.1 Algorithm

Algorithm 1 provides an overview of the proposed PI scheme. This relatively simple algorithm results in policies that inherit the traits discussed in Section 2.3, and thus yields almost optimal admissible policies. Briefly, Algorithm 1 starts with obtaining the simply-connected workspace transformation, in a case where obstacles are present. Then, a number of samples are taken over the boundary, and trajectories are run for the initial policy in order to gather cost data. The cost data are then numerically differentiated and the cost function’s normalized gradient is approximated through a DNN. The policy is finally updated, and the whole scheme is repeated until convergence. Some further details are discussed in the following Subsections.

Algorithm 1 PI ALGORITHM.

  • Given a Workspace W and a convergence threshold $E\in R^{+}$

  **if** W is simply connected **then**
   
$W^{\prime }\leftarrow W$

  **else**
   
$W^{\prime }\leftarrow \text{connect obstacles to boundary}\left(W\right)$

  **end if**
  • Take K∈N samples over the boundary:
  
$p_{k}\in \partial W^{\prime },k\in \left\{1,\cdots \;,K\right\}$

  • Starting from an initial policy $u^{\left(0\right)}\in A\left(W^{\prime }\right)$

  Set i ← 0
  Set converged ←False
  **while** **not**(converged) **do**
   • Compute Cost: V^(i)^ through running trajectories (36) from the initial states $p_{k}\in \partial W^{\prime },k\in \left\{1,\cdots \;,K\right\}$ , gathering data tuples $\left\{x_{j},y_{j},V_{j}\right\},j\in \left\{1,\cdots \;,\bar{N}\right\}$ ,
   • Differentiate the cost numerically,
   • Approximate the Cost Function’s Normalized Gradient with a feed-forward NN,
   • Update the next policy u^(i+1)^ through (16),
   **if** i > 0 **then**
    **if** ‖∇_p_V^(i+1)^ − ∇_p_V^(i)^‖ ≤ E **then**
     converged ←True
    **end if**
   **end if**
   • i ← i + 1
  **end while**
  • Upon convergence i = I the optimal policy is: u^⋆^ ≈ u^(I)^

#### 2.4.2 Normalized gradient approximation

In order to implement (16) the gradient of the cost of the previous policy is required. In Deep RL, usually the cost function is approximated through a DNN from data gathered through trajectories. Note that in Algorithm 1 such a data-gathering process is included. Therefore, a DNN will also be employed in this case. However, since, owing to the deterministic and model-based scheme that is proposed, the relationship between the policy and the cost is explicit through (12) and (13), the **gradient of the cost function** can be employed directly. Still, as can be seen in Eq. 2, obtaining the value for the cost function for a given point inside the workspace requires integrating the trajectory of the robot from the latter unil convergence. While this method can be employed for obtaining a singular value, note that the control laws for the PI method in Eq. 16 require the cost function gradient explicitly, which is not available. Therefore, in order to approximate the gradient, and having obtained values for the surface of the cost function (2) at discrete points ${\hat{V}}_{k,n}=V_{u}\left(p_{k,n}\right)$ we apply an additional step of numerical differentiation, to obtain values for the gradient $\nabla {\hat{V}}_{k,n}$ . The approximation of the gradient field itself, however, presents some challenges. The field admits small values close to the goal position, contrasted to larger values farther away, which results in a poor approximation of the field close to the goal position. This is detrimental for the convergence of the robot to the goal. Hence, we employ the following process: Note that (8) can be written as:

$$\begin{array}{c} {\left(\nabla_{p}V^{\left(i\right)}\right)}^{T}\left(f\left(p\right)+u^{\left(i\right)}\right)=-r\left(p,u^{\left(i\right)}\right)\Rightarrow \\ \|\nabla_{p}V^{\left(i\right)}\|{\hat{e}}_{\nabla_{p}V^{\left(i\right)}}^{T}\left(f\left(p\right)+u^{\left(i\right)}\right)=-r\left(p,u^{\left(i\right)}\right), \end{array}$$ (38)

where ${\hat{e}}_{\nabla_{p}V^{\left(i\right)}}$ denotes the unitary vector along the direction of ∇_*p*_ *V* ^(*i*)^. Consequently, if the unitary vector is known, the norm of the gradient can be obtained through:

$$\|\nabla_{p}V^{\left(i\right)}\|=\frac{\alpha \|p-p_{d}{\|}^{2}+\beta \|u^{\left(i\right)}{\|}^{2}}{\left|{\left(f\left(p\right)+u^{\left(i\right)}\right)}^{T}{\hat{e}}_{\nabla_{p}V^{\left(i\right)}}\right|}.$$ (39)

Therefore, we can normalize the gradient and approximate the **direction** of the field. The field is normalized as follows:

$${\tilde{e}}_{\nabla_{p}V^{\left(i\right)}}\approx \frac{\nabla_{p}V^{\left(i\right)}}{\epsilon +\|\nabla_{p}V^{\left(i\right)}\|},$$ (40)

where *ϵ* is a small, positive constant. If the exact normalization was employed instead of (40), i.e., ${\hat{e}}_{\nabla_{p}V^{\left(i\right)}}=\frac{\nabla_{p}V^{\left(i\right)}}{\left\|\nabla_{p}V^{\left(i\right)}\right\|}$ , then the field would admit singularities inside the workspace. This is owing to the fact that the direction of any field is formally not a two-dimensional unitary vector mapping $\hat{e}\left(p\right):\;R^{2}\mapsto R^{2}$ , but rather maps to a one-dimensional value over the unitary circle $\hat{e}\left(p\right):\;R^{2}\mapsto S^{1}$ . We will however not go into further detail concerning this matter^14^, as the proposed normalization suffices to alleviate any singularities besides for *p*_*d*_, which is however point-like.

In summary, two DNNs are employed^15^, one for each component of the normalized gradient. Subsequently, the norm of the gradient can be computed through (39). The effect of the normalization is to alleviate approximation issues over the workspace, as the training data exhibit significantly less variation, as well as negate the need for a heuristic for weighting the error vector during the training process. In practice, this step produced significantly better and more consistent results than approximating the gradient of the cost or the cost itself, requiring minimal tuning (namely, the DNN layer characteristics) over a variety of workspaces.

#### 2.4.3 Parallel integration scheme

Another significant implementation detail is the parallelizable computation of the trajectories of system (36). Note that each trajectory stemming from an initial point at the boundary can be computed independently. We therefore employ the GPU capabilities of modern machines in order to perform the relevant computation in parallel through a custom parallelized integration scheme, which significantly improves the performance of our method, as it will be presented in the results’ section.

### 2.5 An atlas for alleviating computation in large workspaces

While the proposed method provides several desirable traits owing to the technical results of Section 2.3, it results in expensive computations. In this section, we propose a scheme for alleviating the computational load and the scaling properties of the method, especially when considering large workspaces. This is heavily inspired by Vlantis et al. (2018a) where an atlas of harmonic maps is employed to improve the computational characteristics of the proposed method.

**FIGURE 6:**
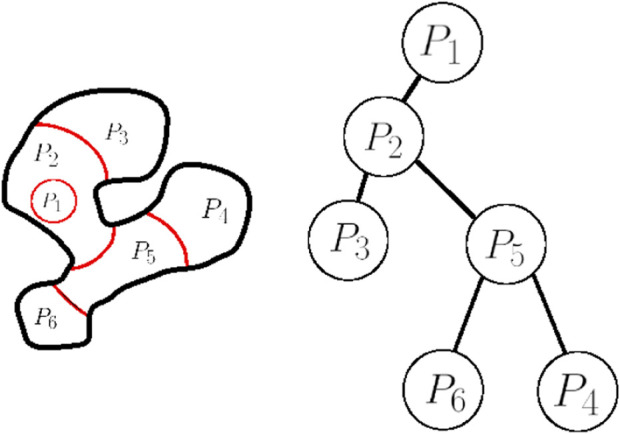
The workspace (left) with its boundary depicted in black lines, and the curves that partition the workspace depicted in red. The corresponding tree is depicted on the right.

Consider an atlas $L=\left\{P_{k}|k\in N_{P}\right\}$ obtained by subdividing the workspace into *N*_*P*_ subsets $P_{k}\subset W^{\prime }$ , i.e., $W^{\prime }={⋃}_{k\in N_{P}}P_{k}$ with $N_{P}=\left\{1,2,\ldots ,N_{P}\right\}$ , where the intersection of two such subsets is at most a one-dimensional Jordan curve, i.e., $C_{k,j}=P_{k}\cap P_{j}=\partial P_{k}\cap \partial P_{j},k,j,\in N_{P}$ , which indicates that the regions intersect at their boundaries in the interior of the workspace. Through proper selection of such subsets, consider a rooted tree structure T=(V,E) such that $V=\left\{P_{k}|k\in N_{P}\right\}$ and $\left(k,j\right)\in E\text{ if f }P_{k}\text{is the parent of }P_{j}$ . Furthermore, the root of T consists of the subset of L that contains the desired position, i.e.,: $P_{\mathrm{root}}≜P_{1}=\left\{P\in L|p_{d}\in P\right\}$ . To prove its existence and further clarify the structure of T as well as the parent-children matrix E , consider the following construction. Given an initial admissible policy *u* ^(0)^, consider *N*_*C*_ = *N*_*P*_ − 1 contours^16^ of the induced cost function *V* ^(0)^:

$$C_{k}^{\prime }=\left\{p\in W^{\prime }|V^{\left(0\right)}\left(p\right)=c_{k}>0,\;k\in N_{C}\right\},$$ (41)

where $N_{C}=\left\{2,\ldots ,N_{C}\right\}$ and which partition $W^{\prime }$ into *N*_*P*_ subsets. Note that each subset will have one or more curves $C_{k}^{\prime }$ as part of its boundary. We denote by *P*_*k*_ the subset for which the cost function *V* ^(0)^ admits the smallest value at the curve $C_{k}^{\prime },\;k\in N_{C}$ (with the exception of the region *P*_1_ which has already been defined). Such an example is depicted in Figure 6. Now consider trajectories of (1) under the policy *u* ^(0)^ starting at a point on the boundary $\bar{p}\in \partial W^{\prime }$ . Note that *V* ^(0)^ is a valid Lyapunov function for (1), while trajectories $p_{u^{\left(0\right)}}\left(t;\bar{p}\right)$ cover the entire workspace (if we consider every $\bar{p}\in \partial W^{\prime }$ , according to Lemma 3). If we denote with $\bar{k}$ the index of the region containing $\bar{p}$ , i.e., $\bar{k}=\left\{k\in N_{P}|\bar{p}\in P_{k}\right\}$ , then by following any such trajectory, a sequence $S_{\bar{p}}=\left\{c_{\bar{k}}\left(\bar{p}\right),c_{s1}\left(\bar{p}\right),\ldots ,c_{\mathrm{sn}}\left(\bar{p}\right)\right\}$ can be constructed, which corresponds to the values of the contours $C_{k}^{\prime },k\in N_{C}$ that the respective trajectory crosses. This sequence is decreasing (owing to *V* ^(0)^ decreasing along a trajectory as a Lyapunov function), and gives rise to the tree structure between the subsets *P*_*k*_. Each branch is formed by considering the set of contours $\left\{C_{\bar{k}}^{\prime },C_{s1}^{\prime },\ldots ,C_{\mathrm{sn}}^{\prime }\right\}$ that any trajectory crosses, on its way to reaching node *P*_1_ which contains the goal position. Therefore, the node *P*_*j*_ admits a parent *P*_*k*_ iff $C_{k}^{\prime }\subset \partial P_{j}$ ^17^. In simply connected spaces this is indeed a tree, as any cycle would imply that the sequence $S_{\bar{p}}$ is not decreasing, leading to a contradiction.

We employ the above tree-decomposition in order to ameliorate the computational performance of the proposed PI scheme, in the following way. Let $\partial W_{k}=\partial W^{\prime }\cap \partial P_{k}$ denote the boundary of the *k*th region that is also part of the boundary of the workspace. Then for any trajectory starting from the point $\bar{p}\in \partial W_{j},j\in N_{C}$ the cost can be split along the trajectory, at its intersection points with the curves $C_{k}^{\prime }$ :

$$V^{\left(i\right)}\left(\bar{p}\right)=\underset{k\in S_{\bar{p}}}{\sum }{\hat{V}}_{k}^{\left(i\right)}\left(\bar{p}\right)+\int_{T_{c_{\mathrm{sn}}\left(\bar{p}\right)}}^{\infty }r\left(\tau ;\bar{p}\right)d\tau$$ (42)

where ${\hat{V}}_{k}^{\left(i\right)}\left(\bar{p}\right)$ denotes the cost that is accumulated over the *k* − th region *P*_*k*_ that corresponds to the initial point $\bar{p}$ :

$${\hat{V}}_{k}^{\left(i\right)}\left(\bar{p}\right)=\int_{T_{k}\left(\bar{p}\right)}^{T_{k+1}\left(\bar{p}\right)}r\left(\tau ;\bar{p}\right)d\tau ,\;k\in S_{\bar{p}}$$ (43)

And $T_{k}\left(\bar{p}\right)$ denotes the time where the trajectory starting from $\bar{p}$ intersects the curve $C_{k}^{\prime }$ . Therefore, to compute a cost along a trajectory, each segment ${\hat{V}}_{k}^{\left(i\right)}\left(\bar{p}\right)$ can be computed independently **in parallel**. Having computed each term ${\hat{V}}_{k}^{\left(i\right)}\left(\bar{p}\right),\;k\in S_{\bar{p}}$ that corresponds to the region *P*_*k*_, the following process is implemented. Starting from the root node, the tree is traversed and at each level of the tree, the appropriate values of the cost function ${\hat{V}}_{j}^{\left(i\right)}\left(C_{j}^{\prime }\right)$ (computed over the child-node region *P*_*j*_ at the common boundary $C_{j}^{\prime }\subset \partial P_{j}$ ) are added to the corresponding trajectories of the parent-node *P*_*k*_. Although this post-processing step is necessarily performed serially, as the information for the cost function propagates along the tree structure, it is numerically cheap.

This further parallelization of the computation of trajectories significantly speeds up the process in large workspaces and it can readily be incorporated with the technical results of Section 2.3.4 to provide a more efficient training scheme, as will become apparent in the results’ section.

## 3 Results

**FIGURE 7:**
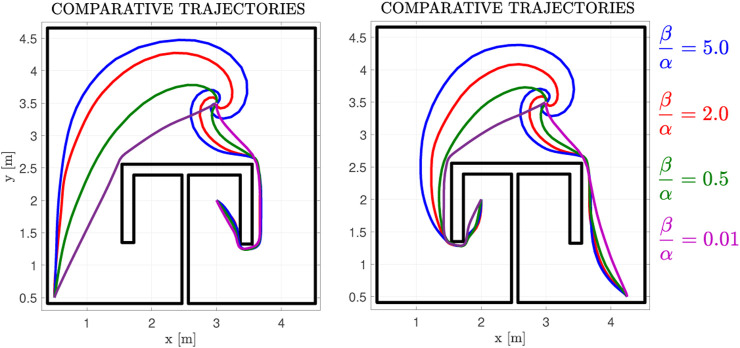
Exemplary Trajectories for a parameter sweep of the input-related cost weight *β* over the state-related cost weight *α*, for the case of the non-linear drift dynamics depicted in Figure 11.

In this section, we demonstrate the validity of the proposed scheme and our technical results, as well as the applicability of our method. We present various workspaces, from the example of a *π*-shaped obstacle as an initial demonstration (transformed into a simply-connected version as discussed in Section 2.2.3), along with more complex maze workspaces. A case for non-linear dynamics is also presented. Furthermore, comparative studies are provided. All results were implemented with MATLAB, version 2021b, running on a PC with an Intel-i7 quad-core processor, with an NVIDIA Geforce GTX-1060 GPU. For all of the presented results (excluding the case of Figure 7) the weighting parameters *α*, *β* were set equal to 1. The distance parameter was chosen as *a* = 0.1 for the *π*-shaped obstacle workspace and as *a* = 0.3 for the maze workspaces. Finally, the employed DNN consists of 3 layers, with sizes $\left(3,5,2\right)$ respectively for all studies.The networks were trained on the cost data using the Mean-Squared Error (MSE) metric for 1,000 epochs.

### 3.1 Proposed method results

#### 3.1.1 Single integrator dynamics

**FIGURE 8:**
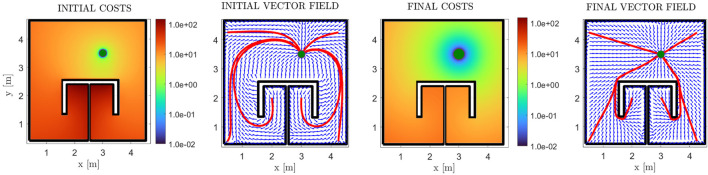
Results for the *π*-shaped workspace, for the case of zero non-linear dynamics. The initial and final cost functions are presented in logarithmic scale (first and third subfigures), along with the respective (normalized) vector fields in blue arrows (second and fourth subfigures). Some exemplary trajectories are also depicted in red lines, with the goal position depicted with a green disk.

**FIGURE 9:**
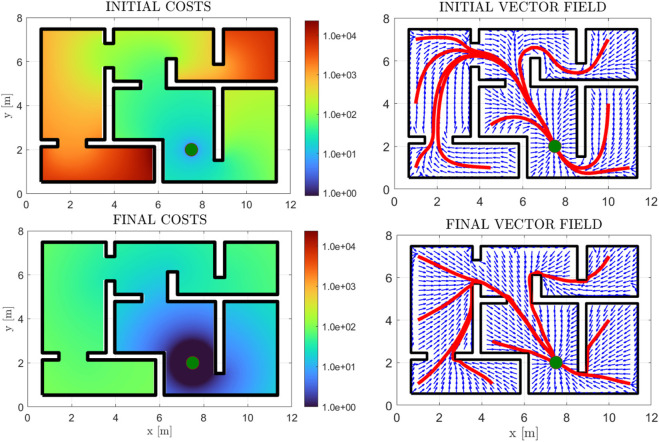
Initial and Final Costs for a maze workspace, in logarithmic scale (left subfigure column). The initial and final normalized vector fields are depicted (blue arrows) (right subfigure column), along with some trajectories (red) and the goal position (green disk).

**FIGURE 10:**
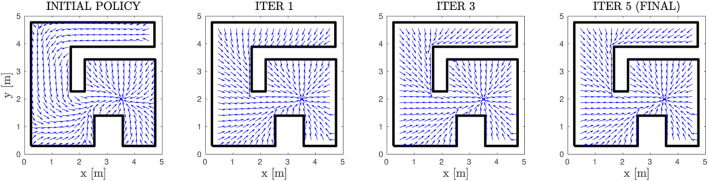
The resulting Vector Fields at different steps of the PI scheme. The normalized velocity field is depicted in blue and the workspace boundary is depicted in black.

First of all, we demonstrate the validity of our method through a simple example of a square workspace with an internal, *π*-shaped obstacle. The initial and simply-connected versions of the workspace are depicted in Figure 2. The comparative cost functions for the initial and final policies are depicted in Figure 8, along with the initial and final (normalized) vector fields, and exemplary trajectories. The improvement of the cost is significant, with the trajectories exhibiting almost optimal path length. We further demonstrate our approach in a more complex environment, the results of which are depicted in Figure 9. The efficacy of the proposed method in decreasing the cost is again demonstrable. We also present “snapshots” of the velocity field for a simple workspace where the effectiveness of the method is apparent in Figure 10. Notably, even after the first iteration of the algorithm, the field already exhibits close to optimal behaviour, which demonstrates the fast convergence of PI methods.

#### 3.1.2 Nonlinear drift dynamics

In this subsection, we present a case where the drift dynamics are non-zero. We consider the case of rotational dynamics with the distance-to-the-goal norm:

$$f\left(p\right)=\|p-p_{d}\|{\left[\sin\left(\theta \right),\cos\left(\theta \right)\right]}^{\text{T}},\;\theta =∠p.$$ (44)

**FIGURE 11:**
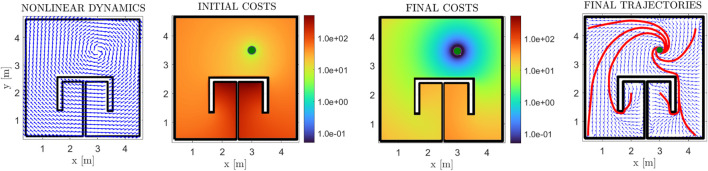
Results for the *π*-shaped workspace, for the case of a rotational vector field for non-linear drift dynamics (left-most plot). The initial and final cost functions are presented in logarithmic scale (second and third subfigures), along with the final (normalized) velocity field in blue arrows (fourth subfigure). Some exemplary trajectories are also depicted in red lines, with the goal position depicted with a green disk.

The normalized dynamics, along with the initial cost function, the final velocity field, and its respective cost are depicted in Figure 11. The initial velocity field is not depicted in this case, as per Eq. 6, it is identical with the one in Figure 8. We note that the costs for the linear and non-linear cases are different, owing to the drift term *f*(*p*). Concerning the final policy of Figure 8 (linear case) the cost of points on the left side behind the *π* obstacle is higher than those on the right side, owing to them being farther away from the goal position. On the contrary, in Figure 11 (non-linear case), the drift term on the left side points towards a direction that “helps” the robot expend less energy, whereas in the right side, the robot has to “overcome” the flow of the drift term to safely navigate towards the goal. This results in the farthest away region behind the obstacle admitting lower cost values. Additionally, in Figure 7, we investigate how trajectories are altered for various ratios *β*/*α*. It is clear that, as the ratio increases, the input-related cost term dominates (2) and thus the algorithm “prioritizes” conserving input energy over converging quickly to the goal. Therefore, the trajectories converge to the goal position slower, while also being “carried along” by the drift dynamics, which results in the spiralling trajectories depicted in Figure 7.

#### 3.1.3 Workspace decomposition

**FIGURE 12:**
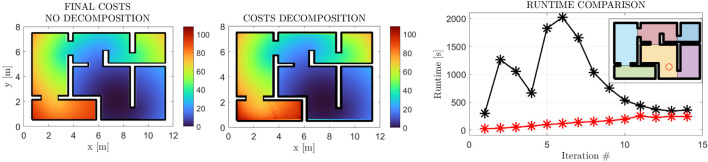
Final Cost Function Comparison with (first subfigure) and w/o (second subfigure) the Decomposition Scheme 2.5. The final cost functions exhibit essentially the same form with a +10% increase at the maximal value. The computational gain is demonstrated in the right-most figure, where the original method’s execution times are depicted in black, while the decomposition scheme’s times are depicted in red. The employed decomposition is also depicted through the shaded regions in the top right corner.

In this subsection, we present the application of the workspace decomposition scheme of Section 2.5. We demonstrate the similarity of the cost functions, along with the employed decomposition and the efficacy of the parallelized scheme in decreasing the computational time of each iteration in Figure 12. The 10% error between the original method and the decomposition scheme (although minimal) can be attributed to the accumulation of the cost function gradient approximation error over the boundaries of the workspace subdivisions. This hypothesis is in accordance with Figure 12, where the error is observed over the remotest subdivision w.r.t. the goal position, where the accumulation of the aforementioned error is expected to have the most impact.

### 3.2 Comparative results

#### 3.2.1 Comparison with continuous methods

**FIGURE 13:**
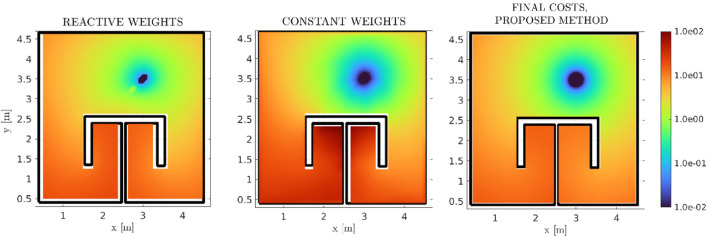
Comparative Final Cost functions between three reactive methods, namely (Rousseas et al., 2020) in logarithmic scale, where the AHPF weights are non-constant functions of the position (first subfigure), a custom, in-house RL scheme which computes the optimal (constant) AHPF weights (second subfigure), and the proposed method respectively (third subfigure). Our method outperforms both previous ones. While it is not self-evident due to the depicted range, the proposed method assumes a maximal value of ≈16 , whereas the method in Rousseas et al. (2020) assumes a maximal value of ≈20 , with similar *relative* cost-value distributions over the workspace.

In order to evaluate the performance of our method, approaches with similar scope and results should be employed. Since the proposed method concentrates on the formal solution of the high-level reactive optimal MP problem (in the form of a velocity vector field), a comparison against the plethora of existing platform-specific DRL approaches would not provide any meaningful comparisons. Therefore, we initially compare the cost function (2) produced by our method against two previous harmonic-based approaches. The results for the reactive, AHPF methods are presented in Figure 13. In the left-most figure, the method by Rousseas et al. (2020) was implemented, where the weights of an AHPF assume a state-feedback form. In the central figure, we present a custom method where a constrained-optimization RL strategy is employed to extract the optimal **constant** AHPF weights. Our method’s cost function is presented again for completeness. It is clear that, although the method by Rousseas et al. (2020) provides descent results, significantly better than the constant weights case, the herein proposed method exhibits a significant overall improvement.

#### 3.2.2 Comparison with sampling-based methods

Subsequently, we employ the length of the produced trajectories, as a metric to compare against two approaches, an RRT^⋆^ and a PRM one. RRT^⋆^ serves as a baseline to evaluate the optimality of our method, as it produces asymptotically optimal results, while PRMs also provide a fair comparison. Nonetheless, note that, even though path length is a valid metric for these methods, the cost function (2) can not be directly evaluated, as they only produce feasible paths and not inputs. In previous works (Rousseas et al., 2020; Rousseas et al., 2021; Rousseas et al., 2022), we have employed RRT^⋆^ planners that include input-space sampling. However, due to sampling over a 4D space, the RRT^⋆^ results presented therein do not consist of fair evaluations against the current method. Nevertheless, we are able to asses the performance of our method even more strictly, as the on-trajectory optimal velocity norm can be computed analytically for any trajectory. More specifically, consider the HJB equation (for the single integrator case):

$${\left(\nabla_{p}V\right)}^{\text{T}}\left(ve_{v}\right)=-\alpha {\left\|p-p_{d}\right\|}^{2}-\beta v^{2},$$ (45)

where $e_{v}\in R^{2}$ denotes a fixed, position-based direction of the velocity field whereas $v\in R_{+}$ denotes its norm. The optimal control input norm is given by $v^{⋆}=-\frac{1}{2\beta }{\left(\nabla_{p}V^{⋆}\right)}^{\text{T}}e_{v}$ . Finally, combining the above two equations yields

$$v^{⋆}=\sqrt{\frac{\alpha }{\beta }}\|p-p_{d}\|.$$ (46)

Therefore, this norm can be applied over the RRT^⋆^/PRM-obtained trajectories to evaluate the global optimality of our method. Note however, that due to the nature of SBMs, the above globally optimal solution produces discontinuous velocity vectors along non-smooth paths, as the latter consist of quasi-linear segments.

We therefore present some exemplary trajectories for the workspace with the *π*-shaped obstacle (against RRT^⋆^), as well as for one of the previously presented complex workspace of Figure 9 (for both sampling-based methods). The results are summed up in Tables 1 and 2. In both cases, 50 trials were carried out to achieve statistical significance for the SBMs. Notably, we conclude that our method exhibits mostly reduced or identical path lengths to the SBM’s ones. Taking into account that, first of all, in our method the field’s norm and direction were concurrently optimized, along with the fact that our method produces a continuous field over the **whole** workspace (in contrast to the RRT^⋆^’s single, non-smooth trajectories), our method can be considered successful in providing globally optimal solutions in the herein presented examples w.r.t. path length.

**TABLE 1:** Comparative results, RRT^⋆^ vs. Proposed Method.

*π*-shaped workspace								
$\bar{p}$	Cost				Path Length [m]			
	Ours	RRT^⋆^			Ours	RRT^⋆^		
		Min	Mean	Max		Min	Mean	Max
[2,2]^*T*^	13.5	**13**	14.3	16.4	4.09	**4.05**	4.31	4.78
[3,2]^*T*^	9.8	**9.3**	10.4	11.4	3.49	**3.39**	3.67	3.96
[0.5,0.5]^*T*^	**16**	16.2	16.7	17.8	**4.1**	4.15	4.25	4.46
[0.5,4.2]^*T*^	**6.8**	6.9	7.1	7.4	**2.6**	2.65	2.71	2.79
[4.2,0.5]^*T*^	**10.4**	10.8	11.1	11.8	**3.27**	3.33	3.42	3.56
[4.2,4.2]^*T*^	**1.7**	2.1	2.2	2.5	**1.44**	1.46	1.5	1.62

The bold values denote the minimum value among the corresponding values.

**TABLE 2:** Comparative results, PRMs and RRT^⋆^ vs. Proposed Method.

Complex workspace														
$\bar{p}$	Cost							Path Length [m]						
	Ours	RRT^⋆^			PRMs			Ours	RRT^⋆^			PRMs		
		Min	Mean	Max	Min	Mean	Max		Min	Mean	Max	Min	Mean	Max
[10,7]^*T*^	**56.1**	56.18	57.67	59.77	58.72	63.65	71.63	8.7	**8.69**	8.84	9.13	8.91	9.57	10.48
[1,7]^*T*^	**66**	73.67	74.20	75.38	73.77	77.15	85.54	**8.49**	8.71	8.77	8.89	8.73	9.05	9.67
[1,4]^*T*^	**67**	73.24	74.91	77.73	74.4	78.67	87.35	**8.78**	9.06	9.18	9.34	9.09	9.6	10.39
[1,1]^*T*^	**91.25**	97.82	99.77	104.16	99.62	105.21	115.84	**11.3**	11.39	11.52	11.67	11.51	12.04	12.93
[4.5,3]^*T*^	**10.1**	10.3	10.6	11.6	11.59	12.97	19.59	**3.12**	3.40	3.44	3.555	3.41	3.78	4.57
[10,4]^*T*^	13.01	**12.17**	12.81	13.66	13.1	15.58	18.77	4.38	**4.18**	4.27	4.46	4.3	4.9	5.93
[11,1]^*T*^	13.2	13.3	13.4	14	**11.87**	13.39	18.05	3.64	3.65	3.7	3.82	**3.5**	3.96	5.27
[4.5,1]^*T*^	**86**	92.18	94.65	98.48	94.31	101.7	108.57	**11.67**	11.90	12.10	12.34	12.32	12.86	13.4

The bold values denote the minimum value among the corresponding values.

Concerning the cost function (2), in Tables 1 and 2 we present comparative cost function values for the *π*-obstacle and complex maze workspaces. Our method produces mostly reduced cost function values compared to the best results from the SBMs, with few exceptions where the cost is nevertheless closely matched. Most importantly, comparing with the median or mean values of these methods, our method is consistently superior. Notably, as the initial position is placed farther from the goal position, our method produces significantly better results, as demonstrated in Table 2. Since our method produces similar or improved path lengths, compared to two SBMs, while also outperforming the latter when the analytically computed optimal velocity norm was applied, we conclude that our method produces close to the globally optimal navigation vector field in the herein presented results. We furthermore note that upon convergence, the velocity norm produced by our method is indeed the optimal one described in (46), as scaling the field appropriately did not alter the results.

Finally, we compare the proposed method for the case of nonlinear dynamics depicted in Figure 11. In Table 3, the cost function values are shown for 10 trials of the SST^⋆^ method (Li et al., 2016) for the six initial points of Figure 11. Non parametric statistical values—minimum, median, maximum values—as well as the mean values are depicted. Our method outperforms the latter by a large margin. This is most likely due to the method being able to plan most effectively for dynamics that are **not** position-dependent (such as unicycle models, Dubins car, etc.) in contrast to the challenging case of position-dependent dynamics, where the SBM is not able to plan a highly optimal tree, producing highly jagged paths. This effect demonstrates the advantages of reactive global planning, where the approach is able to extract a close-to-optimal cost function. Finally, the proposed method is continuous and thus guarantees the applicability of the approach, whereas the SST^⋆^ method performs numerical integration for computing the cost-to-go as well as ensuring safety, which might introduce instabilities or safety violations in practice, in some cases.

**TABLE 3:** Comparative cost values between the proposed method and SST^⋆^.

		*p*_1_	*p*_2_	*p*_3_	*p*_4_	*p*_5_	*p*_6_
SST^⋆^	min	53.7	60.7	46.5	63	33.6	31.3
	mean	78.85	97.7	51.1	76.3	45.3	45
	median	84.9	104.2	50.6	77.6	45.8	47.1
	max	99.3	126.3	56.3	83.5	50.5	51.5
proposed method		21.6	33.7	23.2	9.5	2.7	24.5

### 3.3 Gazebo simulation results

In order to demonstrate the applicability of the proposed scheme, the herein proposed method was employed in a realistic, high-fidelity simulation environment, through the Gazebo (Koenig and Howard, 2004) simulation environment. A simple maze environment was constructed, and the Kuka YouBot (Bischoff et al., 2011) holonomic robot was chosen as the mobile platform, owing to its omni-directional wheels^18^. The workspace’s boundary was augmented during the optimization process so as to account for the robot’s dimensions. The robot is equipped with the Robot Operating System (ROS) (Quigley et al., 2009), and the proposed controller was implemented in MATLAB. Communication between MATLAB and ROS was established through the ROS Toolbox. Since the method is off-line and in the context of known-workspaces, the robot’s odometry, with no additional localization, was employed. Even through the position estimate is not highly accurate, owing to the reactive nature of the proposed scheme, planning proved to be robust enough to always prove successful in this challenging condition. It is evident that a more accurate position estimate (such as with SLAM) would only improve our method’s performance in realistic conditions.

**FIGURE 14:**
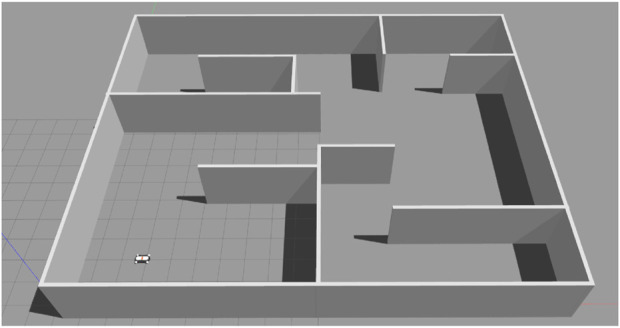
The robot’s workspace in the Gazebo simulation environment, along with the YouBot holonomic robot model.

**FIGURE 15:**
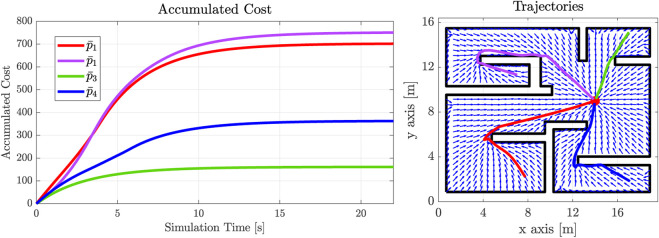
Accumulated on-trajectory cost (left figure) along with the workspace (black line) and four trajectories (right figure). The normalized velocity field is also depicted through blue arrows. The final goal position is placed at [14,9]^T^.

The synthetic workspace, along with the YouBot model, are depicted in Figure 14. The resulting trajectories for four starting configurations, along with the evolution of the on-trajectory cost function (2), are depicted in Figure 15, along with the normalized velocity vector field, for a single final desired position. The cost function parameters were set as *α* = 1.0 and *β* = 40, in order for the robot to move at a “realistic” speed towards the goal (we remind the reader that these parameters are chosen according to design specifications). As can be evidenced by the convergence of the cost function in Figure 15, the robot converged to the final position at approximately 25[*s*] max. A video of the simulation is available through the hyperlink: https://vimeo.com/794492675.

## 4 Discussion

It has been demonstrated, through the aforementioned results, that the proposed method successfully tackles the reactive optimal MP problem. The trajectories demonstrate close to globally optimal behaviour, with advantageous path length and velocity profile. Notably, while the metric (2) does not optimize for the path length directly, we have shown how our method produces nearly optimal path lengths. Notice that the faster way to converge to the desired position with minimal energy expenditure is indeed to follow the minimum-length path while furthermore applying the minimum possible velocity (which depends on the ratio $\frac{\beta }{\alpha }$ ).

With regards to execution times, our method took 6.5 min s and 209 min s for the workspaces of Figures 8, 9 respectively, without the workspace decomposition scheme of Section 2.5. For Figure 12, where the workspace decomposition scheme was employed, the algorithm took 33 min s to converge. In comparison, for the exemplary trajectories for 50 trials of the RRT^⋆^ method, the execution times were 7 min s (1.5 s per trajectory) and 70.4 min s (10.6 s per trajectory) for Figures 8, 9 respectively. The total time for all trajectories was calculated in order to provide a fair comparison due to the following reasons: 1) the 50 trials resulted in the non-parametric statistical results of Tables 1 and 2, therefore, running fewer trajectories would not necessarily yield the minimum values depicted therein, 2) as our method produces results for any initial position, several RRT^⋆^ trajectories are employed to cover the whole workspace (even though we would like to point out that 8 trajectories are still not sufficient for full workspace coverage). Obtaining additional trajectories through the RRT^⋆^ method would require re-running the latter. Our approach, owing to the reactive formulation, provides a reactive solution for **any** starting configuration. A different way to view the above comparison rests on treating the total 400 starting-ending SBM positions as being spread out over the entire workspace, thus resulting in quasi-similar global navigation as in the case of continuous methods. If spread out uniformly over a rectangle of dimensions 11 × 7 [*m*]^2^ (which covers an area of approximately 77[*m*]^2^), the 400 trajectories yield a distribution of approximately 5.2 initial positions per [*m*]^2^. Hence, the initial points would be distributed with a resolution of 2.2 points per meter for each dimension, which is a reasonable coverage for practical applications. Nevertheless, this would emphatically not yield results as satisfactory as those depicted in Table 2, as the above analysis implies one single trajectory per initial point. Nevertheless, SBMs still prove faster in cases where only a single trajectory is required.

While the method proposed in this work exhibits some very promising results, several limitations persist. The main one relates to the curse of dimensionality that plagues PI-related methods, where exhaustive sampling is needed. Indeed, as the number of dimensions grows, the complexity of both the data-gathering and the cost function approximation steps would scale exponentially. Future research efforts will concentrate on ameliorating this aspect of the algorithm, with possible extensions including approximate guaranteed solutions (Jiang et al., 2016) and/or Off-Policy formulations.

Additionally, we intend to extend our work to more complicated state-spaces such as considering orientations and higher-order dynamics. Finally, the computational aspect of our work can be ameliorated.

### 4.1 Future work

We intend to expand the scope of the results in two main ways. First of all, we aim to provide a solution that optimizes the field for any final desired position, in contrast to the present method, where a solution is obtained for a single goal position. Another important aspect is treating unknown robot dynamics with provable guarantees. We further posit that this framework can be easily extended to consider saturated inputs, which would significantly enhance the applicability of the method, as the current formulation necessitates large inputs. Additionally, the framework could be extended for second-order mechanical systems, which would cover a variety of robotic platforms. Finally, the most important future direction that we intend to pursue is to address the existence of obstacles.

## Data Availability

The original contributions presented in the study are included in the article/Supplementary Material, further inquiries can be directed to the corresponding author.

## Footnotes

1 A disk robot can also be considered by applying a workspace transformation that inflates the workspace boundaries: $\partial W^{I}=T\left(\partial W\right)$ where *T*(*z*) = *z* + *Rn*(*z*), $R\in R_{+}$ denoting the robot’s radius and *n* denoting the inwards-pointing vector that is normal to the boundary at the point z∈∂W .

2 While in the following Results section we have provided examples of polygonal workspaces, the proposed method can also be employed for smooth boundaries.

3 This definition ensures that under the control law *u* the robot does not collide with the workspace boundary at any point along all trajectories (for any initial position $\bar{p}$ ).

4 Non-reactive methods are unsuitable due to cost function non-smoothness.

5 Note that in case where the drift term is zero, the velocity *v*(*p*) and the input *u*(*p*) are identical.

6 Even though the method by Rousseas et al. (2022) is employed in the context of unknown workspaces, the framework can be trivially extended to fully known ones by considering the entire boundary as having been sensed by the robot’s sensors.

7 Note that nothing prohibits the vector field *v* exhibiting saddle points exactly at the boundary ∂W , however since the position of the robot lies in the interior of W this does not pose any practical or technical challenges.

8 The term “stable” relates to stability w.r.t. the equilibrium point, not the goal position.

9 Note that, by construction, the vector field (5) has a sink at *p*_*d*_, therefore the case where $p_{d}=p_{i},\;i\in I_{s}$ needs not be considered.

10 If we assume the opposite, then either the goal is not a global minimum of the field, or the field is not safe at the boundary, leading to a contradiction.

11 Successive Approximation as employed by Abu-Khalaf M. and Lewis FL. (2005) can be understood to be a PI scheme.

12 The use of the English letter *a* here is not to be mistaken for the Greek letter *α* in (3a).

13 If the projection were indeed equal to zero, then by the definition of the correction term (19), the robot’s velocity would assume a zero-value, thus leading to lack of convergence.

14 In a few words, we formally approximate two 2-dimensional discontinuous manifolds embedded in $R^{3}$ , whereas in reality we should be approximating a continuous, two-dimensional manifold embedded in $W^{\prime }\times S^{1}$ , the ambient space of which is homeomorphic to the solid torus $D\times S^{1}$ . Be that as it may, such a technical detail is considered outside the scope of this work, and will thus be left untreated at this stage.

15 While a single, two-output DNN can also be employed, in practice we found that it resulted in poorer approximation of the normalized gradient.

16 The choice of a subdivision according to contours of the cost function is not unique, as topologically homeomorphic 2-dimensional curves can also be considered. The complement set of each subdivision however (i.e., $P_{k}^{\prime }≜W^{\prime }-P_{k}$ ) should be forward time invariant. Put simply, this means that any trajectory should not re-enter the set *P*_*k*_ once it leaves the latter, as this would give rise to ambiguity w.r.t. the computation of the total cost, as it will become apparent in the sequel.

17 The parent/child relationship is independent of which trajectory starting in any subset *P*_*k*_ is considered, as all trajectories can be shown to exit *P*_*k*_ by crossing the curve $C_{k}^{\prime }$ , according to Lyapunov arguments.

18 The proposed scheme can also be applied for non-holonomic platforms, owing to the reactive velocity field, through employing a low-level tracking controller.
